# Exploring the feasibility of tensor decomposition for analysis of fNIRS signals: a comparative study with grand averaging method

**DOI:** 10.3389/fnins.2023.1180293

**Published:** 2023-08-10

**Authors:** Jasmine Y. Chan, Murtadha D. Hssayeni, Teresa Wilcox, Behnaz Ghoraani

**Affiliations:** ^1^Department of Psychology, Florida Atlantic University, Boca Raton, FL, United States; ^2^Department of Computer and Electrical Engineering and Computer Science, Florida Atlantic University, Boca Raton, FL, United States; ^3^Department of Computer Engineering, University of Technology, Baghdad, Iraq

**Keywords:** functional near-infrared spectroscopy, tensor decomposition, canonical polyadic decomposition, Tucker decomposition, signal analysis

## Abstract

The analysis of functional near-infrared spectroscopy (fNIRS) signals has not kept pace with the increased use of fNIRS in the behavioral and brain sciences. The popular grand averaging method collapses the oxygenated hemoglobin data within a predefined time of interest window and across multiple channels within a region of interest, potentially leading to a loss of important temporal and spatial information. On the other hand, the tensor decomposition method can reveal patterns in the data without making prior assumptions of the hemodynamic response and without losing temporal and spatial information. The aim of the current study was to examine whether the tensor decomposition method could identify significant effects and novel patterns compared to the commonly used grand averaging method for fNIRS signal analysis. We used two infant fNIRS datasets and applied tensor decomposition (i.e., canonical polyadic and Tucker decompositions) to analyze the significant differences in the hemodynamic response patterns across conditions. The codes are publicly available on GitHub. Bayesian analyses were performed to understand interaction effects. The results from the tensor decomposition method replicated the findings from the grand averaging method and uncovered additional patterns not detected by the grand averaging method. Our findings demonstrate that tensor decomposition is a feasible alternative method for analyzing fNIRS signals, offering a more comprehensive understanding of the data and its underlying patterns.

## 1. Introduction

The use of functional near-infrared spectroscopy (fNIRS) has grown exponentially over the last 20 years due to advances in instrumentation, software, and headgear design. An advantage of fNIRS, as a neuroimaging technique, is that the datasets produced are rich in information with thousands of time samples from multiple channels across conditions and subjects. One of the challenges for researchers is to implement tools for analyzing the fNIRS signal that utilizes this information. Other neuroimaging techniques, such as electroencephalography (EEG), have identified tools like the tensor decomposition method (Mørup et al., 2007; Weis et al., 2009; Dauwels et al., 2011; Vanderperren et al., 2013; Matic et al., 2014) that can optimize their datasets (i.e., find the main patterns emerging in the signal without losing information about the temporal dynamics and spatial configuration). With some methods, changes in cortical response are averaged across time and space, resulting in a loss of information about how the response changes across time and for which areas of the brain. In contrast, tensor decomposition can maintain and reveal these changes across times in the specified area. Additionally, in instances where the time used for analysis is unknown (e.g., not a well-established paradigm), the tensor decomposition method is an alternative to manually testing multiple time periods by hand. However, the effectiveness of tensor decomposition in fNIRS signal analysis has not been investigated yet.

The grand averaging method is a commonly used approach to analyze the fNIRS signal, particularly for identifying group differences in the brain’s hemodynamic response across test conditions. It starts by averaging the changes in the hemoglobin across the time window of interest (TOI) and region of interest (ROI) and then uses a statistical test to identify significant differences; however, there are two main limitations. First, it requires assumptions about the TOI and ROI, which can be limiting if there is no prior knowledge about possible locations in time or space (e.g., using a novel paradigm). Second, averaging across temporal and spatial modes for data reduction may result in a significant loss of information about the temporal and spatial aspects of the hemodynamic response. This may lead to missing important TOIs and ROIs when studying significant differences in the brain’s hemodynamic response across conditions. Another fNIRS signal analysis approach that has been gaining popularity is the general linear model (GLM) (Tak and Ye, 2014; McCullagh and Nelder, 2019; Pinti et al., 2019; von Lühmann et al., 2020). The GLM aims to model the relationship between the fNIRS signals and experimental conditions. The GLM does not make assumptions about the shape of the response; however, it assumes that the fNIRS signal is linear and Gaussian, which may not always be the case.

In this study, the objective was to improve the analysis of multidimensional fNIRS data. The proposed fNIRS signal analysis method is on tensor decomposition, a powerful signal processing and analysis method for handling multidimensional data. It examines the interaction between three or more modes of the signal, such as temporal, spatial, spectral, and subject. The method decomposes the signals into components from each mode to represent the underlying dynamics of the brain across modes (Dauwels et al., 2011; Cong et al., 2015; Rabanser et al., 2017; Wang et al., 2018). Statistical tests can be used on these components to select the ones that indicate significant differences in the hemodynamic response across conditions. These selected components are combined to determine the TOI and ROI representing the significant temporal and spatial differences across conditions.

One of the main advantages of the tensor decomposition method is that it can reveal patterns emerging from the data without making predefined assumptions about the patterns. It has been used in many applications, such as in EEG (Latchoumane et al., 2012; Cong et al., 2013, 2014) and functional magnetic resonance imaging studies (Andersen and Rayens, 2004; Han et al., 2021). Additionally, this method can investigate the interactions between three or more modes of the hemodynamic response (e.g., temporal, spatial, and subjects) without the need for averaging the hemodynamic response over each mode or using a predefined TOI window and ROI. This method can provide a more comprehensive and accurate analysis of the fNIRS signals.

We hypothesized that the tensor decomposition method could identify TOIs and ROIs that significantly differ across conditions without any presumptions about the possible TOIs or ROIs. Two previously collected fNIRS datasets were formulated into tensors to test this hypothesis. Specifically, we used two datasets of hemodynamic responses that were collected from infants as they watched distinct events in a puppet-stage apparatus (Biondi et al., 2016, 2021) and used two different tensor decomposition techniques, canonical polyadic decomposition (CPD) and Tucker decomposition (TD). The CPD and TD were followed by analysis of variance (ANOVA) to identify the TOIs and ROIs that indicated significant differences in hemodynamic responses across conditions. Bayesian analyses were also used on mean hemodynamic response values from the identified TOIs and ROIs to understand the interaction effects. Additionally, we evaluated the usefulness of the tensor decomposition method in the fNIRS field by investigating whether this advanced signal analysis method can replicate the main findings obtained from the grand averaging method and provide additional insights and information that the grand averaging method might have missed due to its limitations.

## 2. Materials and methods

### 2.1. Datasets and data processing

Two fNIRS datasets were used to investigate the performance of the proposed tensor decomposition method for fNIRS signal analysis. The datasets were collected by Biondi and colleagues (Biondi et al., 2016, 2021) to identify cortical structures that support infants’ processing of different types of events. In each of the two datasets, the two types of entities, human/social and nonhuman/mechanical, were crossed with the two types of action sequences to form four event conditions. For both datasets, the studies were conducted with the parent’s written consent and in accordance with the Institutional Review Board at Texas A&M University and Florida Atlantic University.

The first dataset (Biondi et al., 2016), referred to as the Human Hand/Mechanical Claw dataset, utilized a 2 (entity type) × 2 (action sequence) between-subjects design, where 70 infants (29 female) aged six to ten months observed a test event in which a human hand or a mechanical claw (entity type) used a tool in a way that was either functional or nonfunctional (action sequence). Infants in each of the four conditions observed 12 trials of the test event. The second dataset (Biondi et al., 2021), referred to as the Social/Mechanical Interactions dataset, utilized a 2 (entity type) × 2 (action sequence) mixed-model design with entity type (social or mechanical) as the within-subjects variable and action sequence (interaction or no interaction) as the between-subjects variable. This dataset consisted of data from 36 infants (13 females) aged six to nine months. Specifically, one group of infants (*n* = 18) observed events in which social entities engaged in social interactions and mechanical entities engaged in mechanical interactions. Another group of infants (*n* = 18) viewed events in which social entities moved together but did not interact and events in which mechanical entities moved together but did not interact. Each group of infants observed 12 test trials, consisting of a block of six social trials and a block of six mechanical trials. In both datasets, fNIRS data were obtained from 20 channels (10 in each hemisphere) located over bilateral temporal and temporal-occipital cortex (Supplementary Figure S1 for probe placement and geometry). Refer to Section 1 of the Supplementary material for more details on the instrumentation.

The fNIRS data were preprocessed according to Biondi et al. (2016) and Biondi et al. (2021). Refer to Section 1 of the Supplementary material for more details on the preprocessing. Oxygenated hemoglobin data were averaged over trials within each condition and subject to reduce the effect of systemic noise and other outliers, and to create a hemodynamic response function (HRF). The HRF consisted of three-time epochs: baseline (2 s prior to the onset of the stimulus presentation); stimulus presentation (0 s to 15 s for the Human Hand/Mechanical Claw dataset, and 0 s to 12 s for the Social/Mechanical Interactions dataset); and post-stimulus presentation (10 s after the onset of the stimulus presentation). The fNIRS data were collected at different sampling frequencies, 50 Hz for the Human Hand/Mechanical Claw dataset and 25–50 Hz for the Social/Mechanical Interactions dataset, in which the fNIRS data were upsampled to match the number of data points for the tensor decomposition analysis.

### 2.2. Grand averaging method

**Figure 1:**
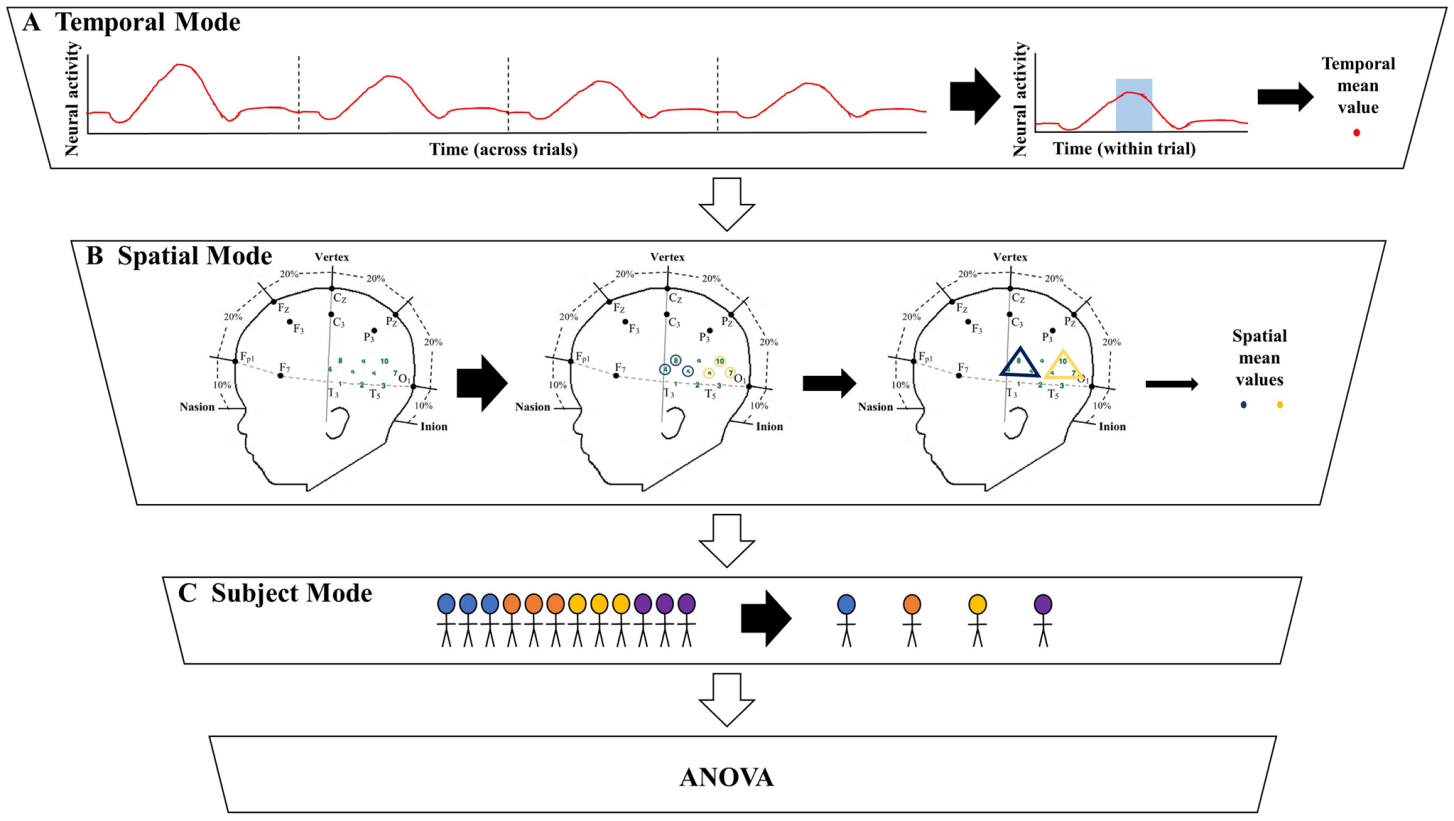
Illustration of the significant amount of data reduction when implementing the grand averaging method before assessing significant differences across conditions. **(A)** To prepare for fNIRS signal analysis, a hemodynamic response function (HRF) is constructed by averaging across multiple trials within a condition. From there the grand averaging method functions by averaging across the temporal mode by using a predefined time of interest (TOI) window to average across, resulting in a single temporal mean value for each channel. **(B)** Then the grand averaging method averages across the spatial mode. For illustrative purposes, there are two regions of interest (ROIs) shown in the triangles. Temporal mean values obtained from channels in the same ROI are averaged, resulting in a single spatial mean value for each ROI per subject. **(C)** The spatial mean values collected from all the subjects are then grouped together by condition to be analyzed with analysis of variance (ANOVA).

**Figure 2:**
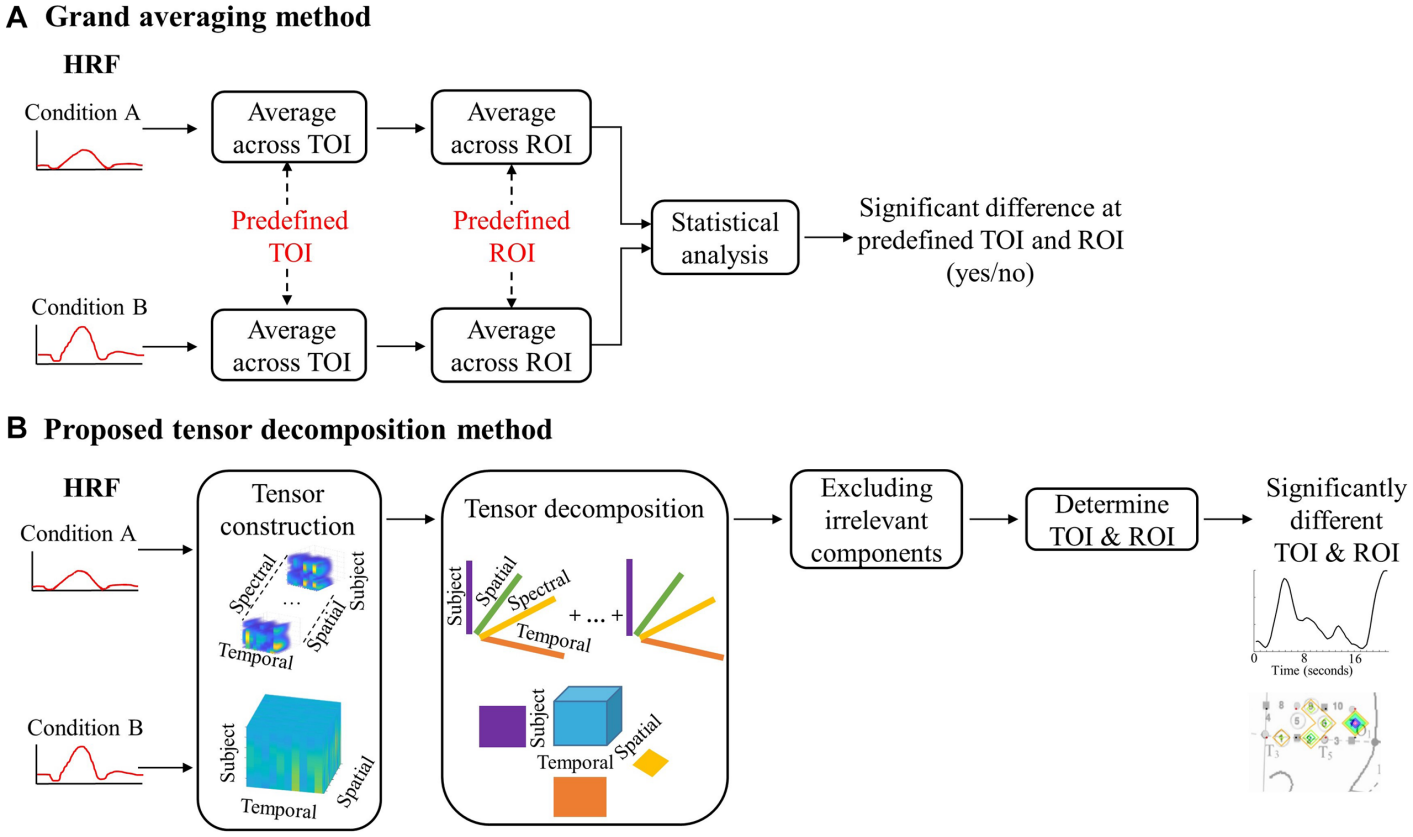
A flowchart depicting the steps for grand averaging and the proposed tensor decomposition method. **(A)** Visual representation of the data reduction with grand averaging. **(B)** Visual representation of the data preservation with the proposed method.

The grand averaging method was applied to the HRF signals from the two datasets by first predefining a TOI window and ROI (Figures 1, 2A). The HRF was then reduced across the temporal mode by averaging the HRF over the predefined TOI window to obtain a single temporal mean value. This process was repeated across the spatial mode by averaging the temporal mean values over channels within the ROI (i.e., averaging channels within an ROI after averaging over the TOI), resulting in a single spatial mean value per ROI. These spatial mean values were then grouped by condition and tested for statistically significant differences using ANOVA. This method allowed for the examination of the temporal dynamics of the hemodynamic response during a specific TOI within a specific ROI. Refer to Section 2 of the Supplementary material for more details.

### 2.3. Proposed tensor decomposition methods

This section explains the process of applying the tensor decomposition method to the HRF. Refer to Section 3 of the Supplementary material for the mathematical notations. To ensure reproducibility, we made the codes public. The codes for the proposed method are publicly available on GitHub (Chan et al., 2023).

#### 2.3.1. Tensor construction

The tensor decomposition method involves creating a four-dimensional array, called a tensor, which includes temporal, spatial, spectral, and subject modes (Kolda and Bader, 2009; Sidiropoulos et al., 2017). The subject mode consists of all subjects in all of the conditions. For the first dataset, it is a between-subjects design with 70 infants. This means that the size of the subject mode is 70 subjects. The data were first transformed into time-frequency representations using Short-time Fourier transform. Only the Fourier Transform coefficients for positive frequencies were used to ensure consistent significant components were revealed after using nonnegative CPD. The nonnegativity constraint only revealed changes in the hemodynamic response, not if the changes were above or below zero activity. This allows for the investigation of significant changes in the response’s spectral behavior; however, our preliminary investigations did not find any differences across conditions on the spectral mode (Hssayeni et al., 2020). Therefore, we created a three-way tensor with temporal, spatial, and subject modes when applying TD as the tensor decomposition method. The tensor was then divided into two separate tensors, one for each hemisphere, to examine differences in the hemodynamic response patterns for the left and right hemispheres. This process is illustrated in Figure 2B, where the top half shows the tensor created for CPD and the bottom half shows the tensor created for TD.

#### 2.3.2. Tensor decomposition

The tensor decomposition method is a technique used to analyze the interactions between multiple modes of a tensor (Hitchcock, 1927; Mørup et al., 2007; Cichocki et al., 2015; Cong et al., 2015) and extract the main components of the underlying complex dynamics (Mørup, 2011). This is done by giving more weight to similar patterns of the signal across the tensor and less weight to the background noise, such as systemic physiology, machine noise, and motion artifacts. The two most popular tensor decomposition techniques, CPD and TD (Cong et al., 2015), were used in this study. CPD decomposed the four-way tensor X into *R* number of components (Carroll and Chang, 1970; Kolda and Bader, 2009; Cichocki et al., 2015; Rabanser et al., 2017; Eq. 1). Each component consisted of the outer product of the four vectors ($u_{r}^{\left(t\right)}$, $\;u_{r}^{\left(f\right)}$, $u_{r}^{\left(c\right)}$, and $u_{r}^{\left(s\right)}$ $\in R^{1\times R}\;)$ which were the temporal, spectral, spatial, and subject modes, respectively.

(1) X
$$\begin{array}{c} \approx I\times_{1}U^{\left(t\right)}\times_{2}U^{\left(f\right)}\times_{3}U^{\left(c\right)}\times_{4}U^{\left(s\right)}\\ \approx \;\sum_{r=1}^{R}u_{r}^{\left(t\right)}{}^{\circ}u_{r}^{\left(f\right)}{}^{\circ}u_{r}^{\left(c\right)}{}^{\circ}u_{r}^{\left(s\right)}\; \end{array}$$

Different symbols were used in the equations to avoid confusion between CPD and TD (Tucker, 1966; Kolda and Bader, 2009; Cong et al., 2015; Rabanser et al., 2017). TD decomposed the three-way tensor Y into a core tensor,G, and $R^{R_{t}\times R_{c}\times I_{s}}\;$number of components from each mode ($A^{\left(t\right)}$, $A^{\left(c\right)},$and $A^{\left(s\right)}$; Eq. 2). The core tensor represents the main underlying patterns by showing how each mode’s components connect (Zubair and Wang, 2013). For the current study, the core tensor consisted of the product of the components, $g\in R^{R_{t}\times R_{c}\times I_{s}},$ from the temporal, spatial, and subject modes, respectively. The number of extracted components, $R_{t}$ and $R_{c}$, was less than or equal to the total number of data points in the according mode, $I_{t}$ and $I_{c}$. Only the number of subject components,$\;I_{s}$, was not decomposed so that ANOVA could be used to identify significant differences across conditions and so that each subject would have a temporal and spatial component that would reveal the TOI and ROI, respectively. That is the information from the subjects mode was not compressed. For example, it is possible that 70 subjects could be represented with 5 components. In the case of the current experiment, the subject mode was not compressed, and 70 components were used to represent the 70 subjects. Also, it was so that each subject would have a temporal, $a_{r_{t}}^{\left(t\right)}$, and spatial, $a_{r_{c}}^{\left(c\right)}$, component that would reveal the TOI and ROI, respectively.

(2) Y$$\begin{array}{l} \approx G\times_{1}A^{\left(t\right)}\times_{2}A^{\left(c\right)}\times_{3}A^{\left(s\right)}\\ \approx \sum_{r_{t}=1}^{R_{t}}\sum_{r_{c}=2}^{R_{c}}\sum_{r_{s}=1}^{I_{s}}g_{r_{t}r_{c}R_{s}}a_{r_{t}}^{\left(t\right)}a_{r_{c}}^{\left(c\right)}a_{r_{s}}^{\left(s\right)} \end{array}$$

The tensor decomposition methods involve identifying the number of components in the data by minimizing the differences between the original and decomposed tensors while balancing accuracy and compression. The optimal number of components was determined by finding the point at which there is a significant decrease in the relative error. For CPD, the number of components, R, extracted should have a reconstruction error rate below 10%. For TD, the same method was used to estimate the number of components, $R^{R_{t}\times R_{c}\times I_{s}}$, that should have been extracted. Here, we used the nonnegative CPD for stability (Cong et al., 2014) and orthogonal TD for unique decomposition (Phan and Cichocki, 2010). The optimization algorithm for computing the CPD and TD was alternating least squares and low multilinear rank approximation, respectively.

#### 2.3.3. Excluding irrelevant components

The temporal, spatial, and spectral components extracted from the tensor decomposition methods were evaluated for relevance to the typical hemodynamic response. Components that did not meet these criteria were excluded from further analysis. Although the exclusion of components started with visual inspections, objective cutoffs were set and applied to all components.

**Figure 3:**
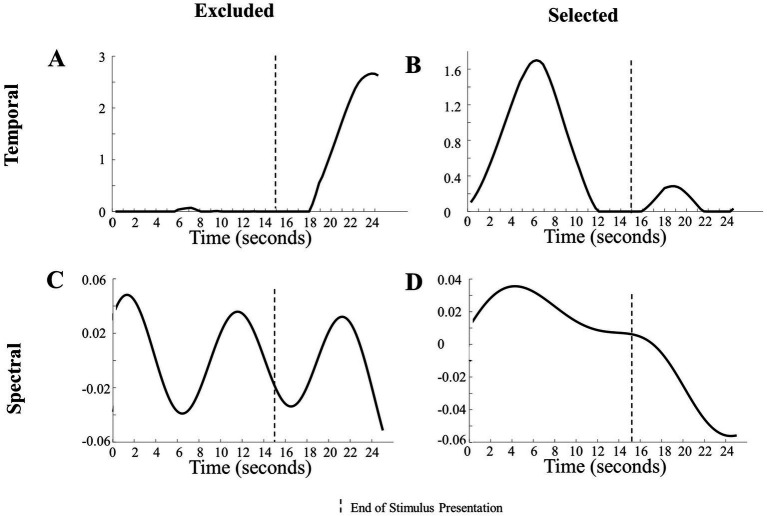
Examination of components in the analysis. **(A)** Representation of a temporal component excluded from analysis due to a lack of change in magnitude during the presentation of the stimulus. **(B)** Representation of a temporal component included in the analysis due to its noticeable changes during the stimulus presentation. **(C)** Depiction of an excluded temporal component with frequencies >0.1 Hz. **(D)** Illustration of an included temporal component with low frequencies <0.1 Hz.

Visual inspection of the temporal components revealed that some components had changes in magnitude mostly during the baseline period. This led us to set objective boundaries for excluding temporal components with a low absolute magnitude during stimulus presentation, as that indicated a lack of hemodynamic response. Specifically, temporal components with a mean absolute value less than 0.01 from 2 s after stimulus onset to the end of stimulus presentation were excluded. This mean of 0.01 cutoff was determined by visually inspecting all components. Figure 3A reveals that the changes in magnitude were during the baseline period (i.e., no visual stimuli were presented). This means that the changes in magnitude from the component reflected the response to the baseline period and not the stimuli being researched.

Objective exclusions of the spectral components were based on prior literature and the length of the paradigm. Spectral components that had high frequencies (> 0.1 Hz in the Human Hand/Mechanical Claw dataset and > 0.5 Hz in the Social/Mechanical Interactions dataset) were excluded, as the hemodynamic response is typically below 1 Hz (Di Lorenzo et al., 2019). Other spectral parameters should be used for adult participants or if the paradigm is a different length.

In the case of CPD, an additional exclusion criterion was determined objectively and applied. Due to the nonunique decompositions, there were variations in the components emerging across the multiple runs (Hssayeni et al., 2020). Temporal and spatial components that had a moderate positive association (*r* > 0.5, α = 0.05) across multiple runs and only including those that consistently emerged. The temporal components that had a moderate positive association were weighted proportionally in the frequency of occurrence across runs and combined. The same criteria were used to identify spatial components that had a moderate positive association (*r* > 0.5, α = 0.05) with other spatial components across runs. See Figure 3 for examples of components that were excluded and included in the analysis based on these criteria.

#### 2.3.4. Determination of TOI and ROI

**Figure 4:**
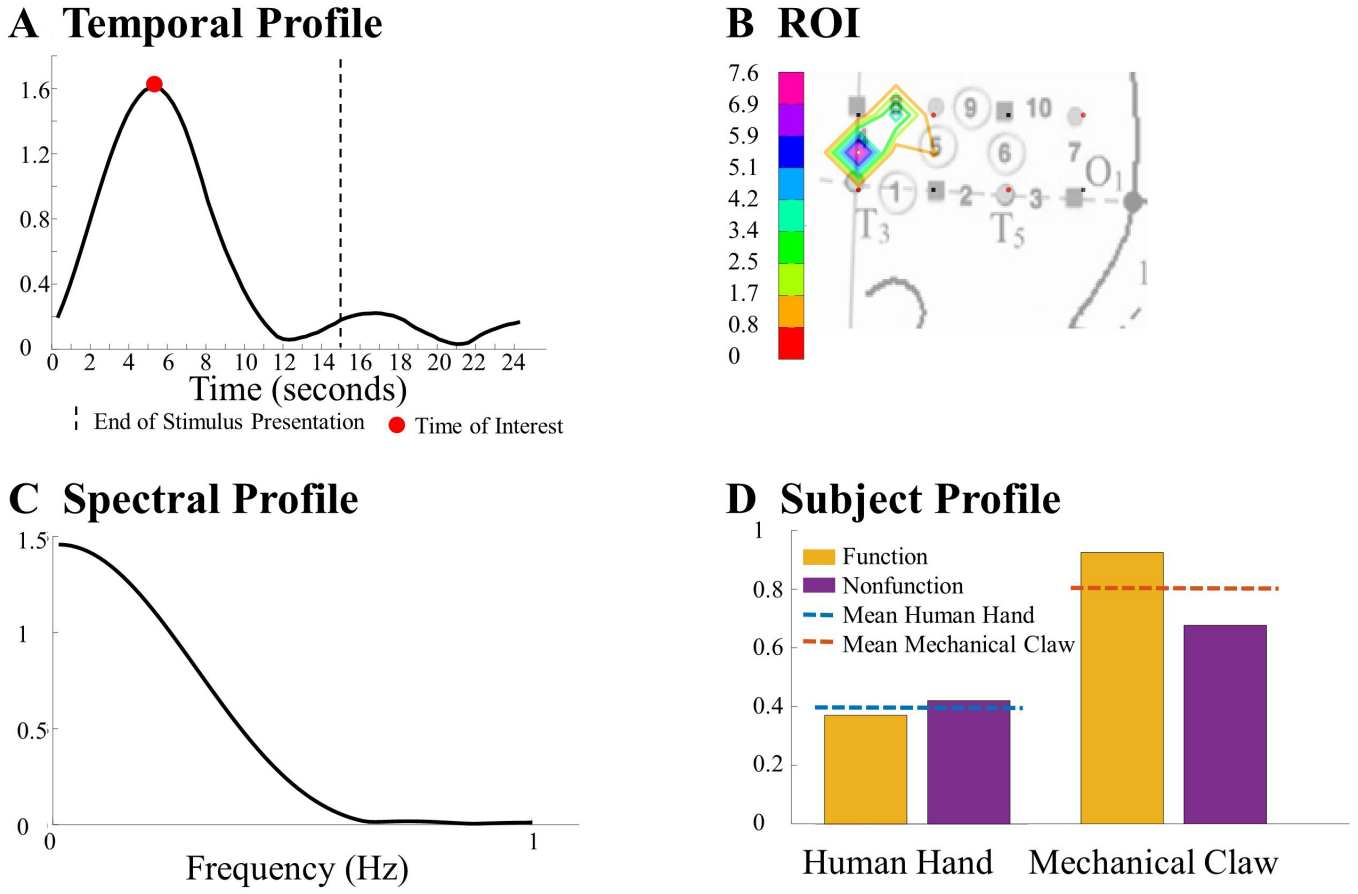
This figure highlights a significant difference observed between the human hand and the mechanical claw from the CPD analysis, including **(A)** a temporal profile of the TOI, **(B)** a ROI map, **(C)** a spectral profile, and **(D)** a subject profile. Further details on this result are in section 3.1.1.

After the exclusion of irrelevant components, ANOVA was applied to the subject components to identify combinations of temporal, spatial, and spectral (for CPD) components that reveal a significant difference across conditions as a function of the entity type, action sequence, or the Entity Type × Action Sequence interaction (α = 0.05). Specifically, a 2 (entity type) × 2 (action sequence) ANOVA was applied to each component of the subject’s mode in the Human Hand/Mechanical Claw dataset, and a mixed-model 2 (entity type) × 2 (action sequence) ANOVA was applied to each component of the subject’s mode in the Social/Mechanical Interactions dataset. The significant temporal, spatial, and spectral (for CPD) components were then summed together in the corresponding significant effects and hemisphere to represent the temporal profile, ROI, and spectral profile (for CPD) to identify response differences across conditions. See Figure 4 for an example of the temporal profile, ROI, spectral profile, and subject profile from CPD that identified a significant main effect of entity type. It is important to note that the temporal profile should not be interpreted as an HRF. The temporal profile and HRF differed in a few ways. The temporal profile represents coefficients from the temporal component of the tensor decomposition. These coefficients reflect the main patterns emerging in the HRF. When tensor decomposition is used, the HRF is represented by combining multiple components. In contrast, the temporal profile is constructed from component(s) that reveal a significant effect. Additionally, given the type of constraint used on the tensor decomposition, the direction of these changes in the HRF (e.g., activation or deactivation) is not reflected. It is because of all these differences; the temporal profile is not on a one-to-one ratio with the HRF. The temporal profile and HRF are similar in that both represent temporal dynamics (i.e., changes in the hemodynamic response across time); however, the direction of this change is not reflected in the temporal profile and the magnitude is not a one-to-one ratio with the HRF. The temporal profile in the current study represents the significant pattern that CPD or TD identified across conditions and was used to determine the TOI. Higher values in the temporal profile identify the point in time (i.e., the TOI) in which there are significant differences in the hemodynamic responses across conditions. If a peak in the temporal profile occurred post-stimulus presentation, it was not considered a TOI. The ROI was identified by the channel(s) that revealed the most prominent difference across conditions. The spectral profile from CPD was used to identify the frequency at which the responses differ based on the manipulation. The subject profile indicates if it is main effect of entity type, action sequence, or the interaction between them.

## 3. Results and discussion

MATLAB (Mathworks, Natick, MA) with the Tensorlab 3.0 (2016) was used to perform the CPD and TD. CPD was run ten times on the left and right hemisphere tensors to account for its nonuniqueness and to reveal all variations (Hssayeni et al., 2020), while TD was run once on the two tensors. Presented below are the results of the grand averaging method compared to the tensor decomposition method.

### 3.1. Human hand/mechanical claw dataset

The CPD extracted 50 components, while the TD extracted 8 temporal components, 10 spatial components, and 70 subject components for the left and right hemisphere tensors. The number of components selected and combined from CPD and TD to represent the changes in hemodynamic response are shown in Table 1, respectively. A total of 500 components were extracted across the ten runs for CPD. The values in Table 1 represent the total number of components that revealed a significant effect out of those 500. For TD, eight temporal and ten spatial components were extracted, resulting in 80 possible combinations from crossing those components. The values in the top row of Table 1B indicate the number of combinations that revealed a significant effect out of those 80. These significant components from CPD and TD were then summed across the corresponding temporal and spatial modes.

**Table 1:** Component selection and combination for the human hand/mechanical claw dataset.

	Left hemisphere			Right hemisphere		
	Main effect of entity type	Main effect of action sequence	Entity type × action sequence interaction	Main effect of entity type	Main effect of action sequence	Entity type × action sequence interaction
A. CPD	27	0	9	77	11	16
B. TD	2 combinations 2 T & 2 S	0 combinations 0 T & 0 S	2 combinations 2 T & 2 S	7 combinations 4 T & 5 S	0 combinations 0 T & 0 S	2 combinations 2 T & 2 S

(A) The total number of components selected to represent the important patterns after performing CPD. A total number of zero indicates that there was not a significant effect. (B) The top values indicate the total number of temporal and spatial component combinations selected to represent the important patterns after performing TD. “T” represents the number of selected temporal components, and “S” represents the number of selected spatial components used to create the combinations. For TD, eight temporal and ten spatial components were extracted, resulting in 80 possible combinations from crossing those components. The values in the top row of Table 1B indicate the number of combinations that revealed a significant effect out of those 80 combinations.

#### 3.1.1. Comparison to grand averaging

**Figure 5:**
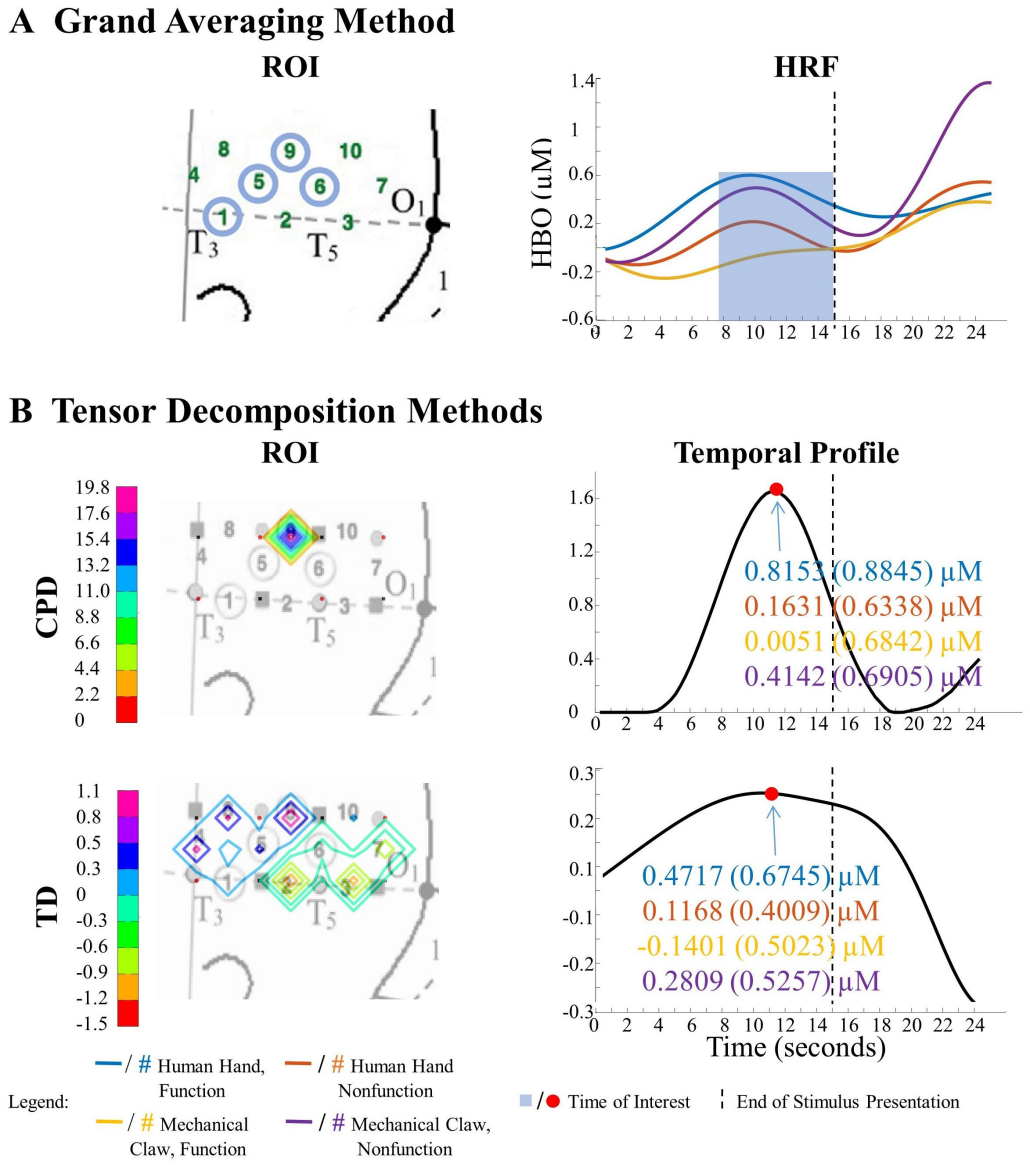
Significant Entity Type × Action Sequence interaction in left hemisphere. **(A)** Grand Averaging Method: There was a significant difference in hemodynamic response across conditions during the function event within the statistically defined channels of the ROI and predefined TOI window in the HRF. **(B)** Tensor Decomposition Method: CPD and TD identified a significant ROI and TOI. The graph displays the mean hemodynamic response and standard deviation for each condition, calculated by averaging 1 s before and after the identified TOI within the ROI. The high values in the ROI and temporal profile indicate the channels and time where there was a difference across conditions.

**Figure 6:**
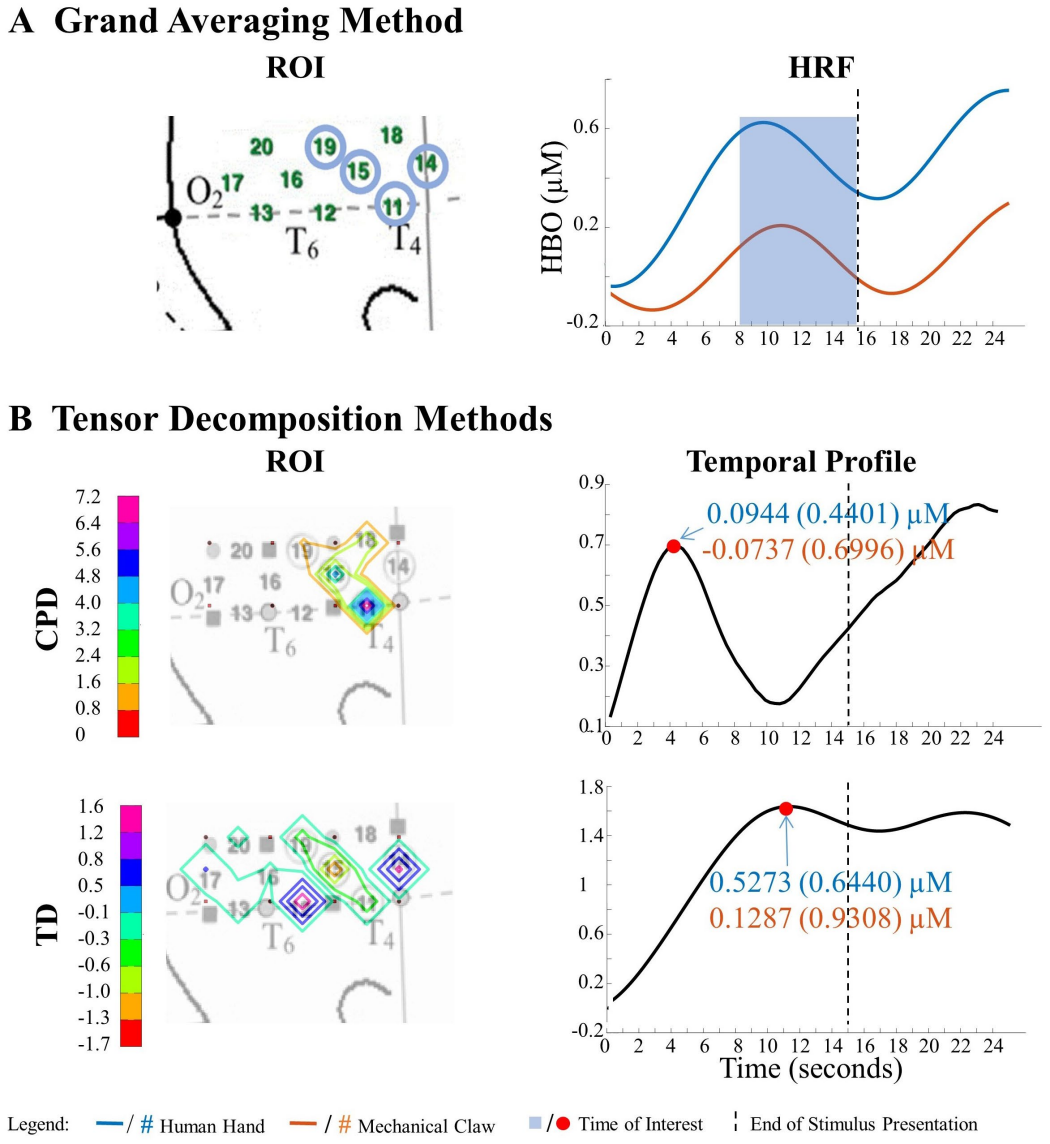
Significant main effect of entity type in right hemisphere. **(A)** Grand Averaging Method: The graph displays the significant difference in hemodynamic response between the human hand and mechanical claw for the statistically defined ROI and predefined TOI window within the HRF. **(B)** Tensor Decomposition Method: CPD and TD identified a ROI and TOI.

**Figure 7:**
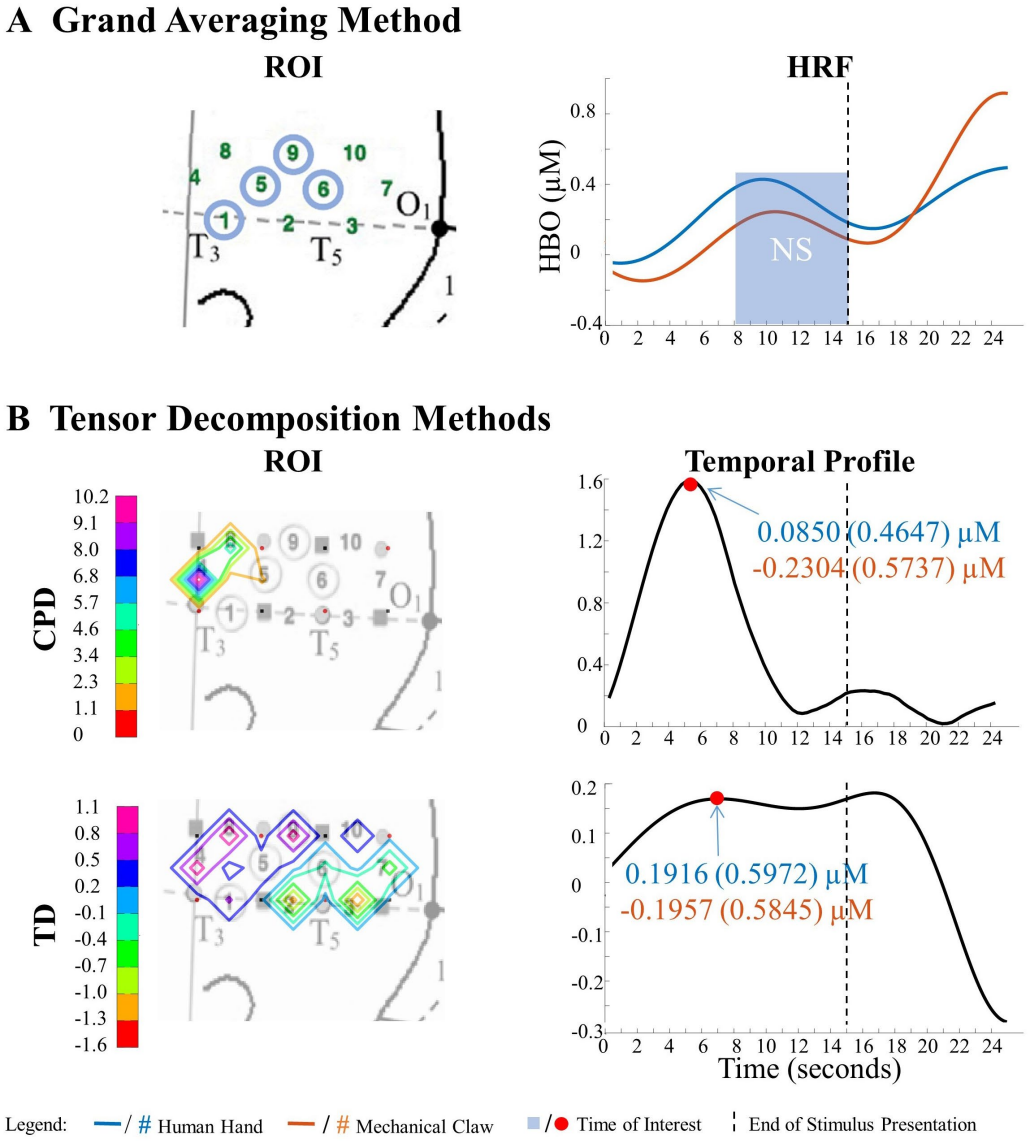
Tensor decomposition method revealed a novel main effect of entity type in left hemisphere. **(A)** Grand Averaging Method: No significant (NS) difference between human hand and mechanical claw was found for the statistically defined ROI and predefined TOI window within the HRF. **(B)** Tensor Decomposition Method: CPD and TD agreed on a novel significant difference and identified a ROI (anterior temporal cortex for CPD and anterior/middle temporal cortex for TD) and TOI (first half and second half of stimulus presentation for CPD and TD, respectively).

**Figure 8:**
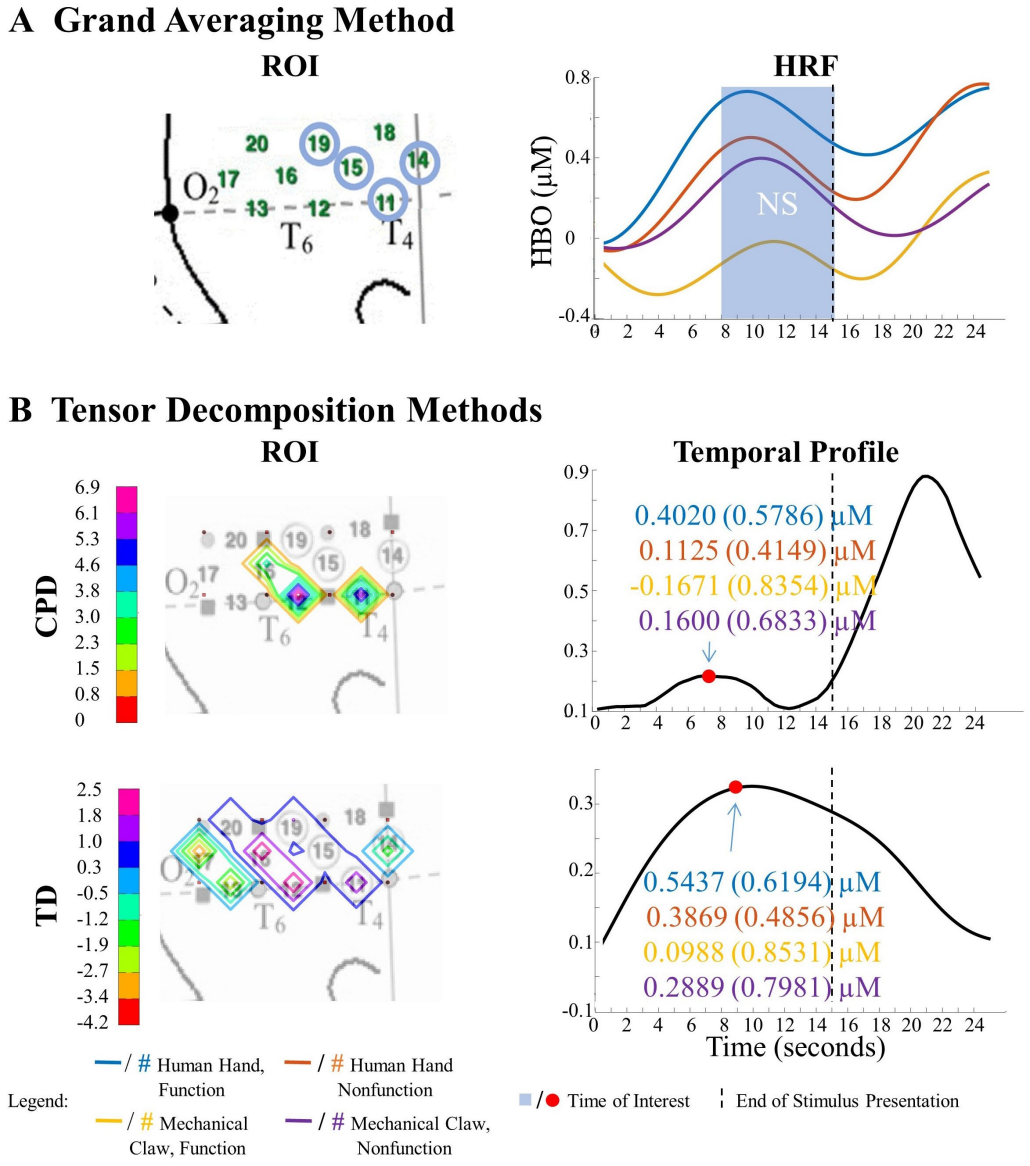
Tensor decomposition method revealed a novel Entity Type × Action Sequence interaction in right hemisphere. **(A)** Grand Averaging Method: No significant (NS) difference across conditions was found for the statistically defined ROI and predefined TOI window within the HRF. **(B)** Tensor Decomposition Method: CPD and TD agreed on a novel significant difference and identified a ROI (inferior temporal cortex for CPD; middle temporal cortex for TD) and TOI (first half and second half of stimulus presentation for CPD and TD, respectively).

The results obtained from the tensor decomposition methods were compared to those obtained from the grand averaging method, as reported by Biondi et al. (2016) (Table 2). Figures 5–8 highlight some of the more interesting patterns identified. An additional pattern found with CPD can be seen in Supplementary Figure S4 and Supplementary Tables S3, S4 of the Supplementary material. It is important to note that the figures show the results from the grand averaging method that reveal the channels in a ROI and the mean HRF obtained when averaged across the channels in that ROI. On the other hand, results from the tensor decomposition methods reveal the significant ROIs and TOIs that emerged from data analysis (i.e., patterns that emerged without imposing preconceived assumptions about the patterns). The values in the ROI represent channels that indicate a difference in the hemodynamic response as a function of the entity type, action sequence, and the Entity Type × Action Sequence interaction. The values in the temporal profile represent the TOI that indicates a difference in the hemodynamic response as a function of the manipulation. Additionally, spectral information from CPD was not included as there were no differences across conditions.

**Table 2:** Comparison of the results obtained using the grand averaging method, CPD, and TD on the Human Hand/Mechanical Claw dataset for both hemispheres.

	Grand averaging	Canonical Polyadic Decomposition (CPD)	Tucker Decomposition (TD)
Main Effect of Entity Type in Left Hemisphere (Figure 7)	NS	channels 4, 5, & 8	channels 4, 8, & 9
Main Effect of Entity Type in Right Hemisphere (Figure 6)	channels 11, 14, 15, & 19	channels 11 & 15	channels 11, 15, & 19
Main Effect of Action Sequence in Left Hemisphere	NS	NS	NS
Main Effect of Action Sequence in Right Hemisphere (Supplementary Figure S4)	NS	channel 15	NS
Entity Type × Action Sequence Interaction in Left Hemisphere (Figure 5)	channels 1, 5, 6, & 9	channel 9	channels 1, 4, 5, 8, & 9
Entity Type × Action Sequence Interaction in Right Hemisphere (Figure 8)	NS	channels 11 & 12	channels 12 & 16

This table includes patterns for entity type, action sequence, and the interaction between entity type and action sequence. “NS” indicates a nonsignificant effect.

##### 3.1.1.1. Tensor reveals similar results as grand averaging

The results of the grand averaging method, CPD, and TD all revealed a statistically significant Entity Type × Action Sequence interaction in the left hemisphere, specifically in the ROI formed by channels 1, 5, 6, and 9 for the grand averaging method, channel 9 for CPD, and channels 1, 4, 5, 8, and 9 for TD (Figure 5; Supplementary Table S1 for *F*-values and *p*-values). Bayesian analyses (Jeffreys, 1961; Kruschke, 2015) were conducted on the mean hemodynamic responses from 1 s before to 1 s after the TOI and ROIs identified with the tensor decomposition methods to identify the source of the interaction (Figure 5 and Supplementary Table S2 for means and standard deviations of the hemodynamic response calculated from the TOI and ROI indicated by grand averaging, CPD, and TD). A greater hemodynamic response was obtained for the human hand, function events than the mechanical claw, function events (all BFs > 13.2). There was no support for the alternative hypothesis when comparing the mean hemodynamic response obtained to the human hand, nonfunction event to that obtained to the mechanical claw, nonfunction event (all BFs < 1). The results of all three methods were consistent, showing a greater hemodynamic response to the human hand than the mechanical claw, but only during functional events.

The grand averaging method, CPD, and TD all revealed a significant main effect of entity type in the right hemisphere (Supplementary Table S1). The results from the grand averaging method identified channels 11, 14, 15, and 19 (Figure 6A). CPD identified a statistically significant main effect in channels 11 and 15 during the first half of the stimulus presentation, and TD identified a statistically significant main effect in channel 14 during the second half of the stimulus presentation (Figure 6B). In all three methods, there was a greater response to the human hand than the mechanical claw, regardless of the sequence (Supplementary Table S2).

##### 3.1.1.2. Tensor reveals additional results to grand averaging

The grand averaging method results showed no main effect of entity type in the left hemisphere (Figure 7A; Supplementary Table S1). In contrast, tensor decomposition methods revealed a significantly greater hemodynamic response to the human hand than the mechanical claw during the first half of stimulus presentation in channels 4, 5, and 8 for CPD and channels 4, 8, and 9 for TD (Figure 7B; Supplementary Table S2).

The grand averaging method did not identify a significant Entity Type × Action Sequence interaction in the right hemisphere (Figure 8A; Supplementary Table S1). However, CPD identified a statistically significant interaction effect in channels 11 and 12 during the first half of the stimulus presentation (Figure 8B; Supplementary Table S1). Bayesian analyses conducted on the mean hemodynamic responses obtained at the TOI and ROI showed substantial evidence for a greater response to the human hand than the mechanical claw during function events (BF = 5.18) but no support for the alternative hypothesis during nonfunction events (BF < 1, Supplementary Table S2). TD identified a significant interaction effect during the second half of the stimulus presentation in channels 12 and 16 (Figure 8B; Supplementary Table S1). However, Bayesian analyses found weak evidence for a greater hemodynamic response to the human hand than mechanical claw during function events (BF = 2.64) and no evidence at nonfunction events (BF < 1, Supplementary Table S2). The mean hemodynamic response obtained at the TOI in both tensor decomposition methods confirmed an Entity Type × Action Sequence interaction.

Overall, findings support using the tensor decomposition method for fNIRS data analysis as it replicated results from the grand averaging method and identified patterns missed by the grand averaging method. The three methods were further compared using a separate dataset to test the efficacy of the tensor decomposition method.

### 3.2. Social/mechanical interactions dataset

The number of components extracted for CPD was 60 per run (i.e., 600 components extracted across ten runs). Table 3 indicates the number of components that showed a significant effect. The core tensor of TD was set to extract 17 temporal components, 10 spatial components, 72 subject components for the left hemisphere tensor and 23 temporal components, 10 spatial components, 72 subject components for the right hemisphere tensor. The top row of Table 3B shows the number of combinations of temporal and spatial components that revealed a significant effect.

**Table 3:** Component selection and combination for the Social/Mechanical Interactions dataset.

	Left hemisphere			Right hemisphere		
	Main effect of entity type	Main effect of action sequence	Entity type × action sequence interaction	Main effect of entity type	Main effect of action sequence	Entity type × action sequence interaction
A. CPD	23	125	10	16	99	27
B. TD	89 combinations 14 T & 10 S	86 combinations 15 T & 9 S	88 combinations 14 T & 10 S	118 combinations 19 T & 9 S	118 combinations 20 T & 10 S	110 combinations 19 T & 10 S

(A) The total number of components selected to represent the important patterns after performing CPD. (B) The top values indicate the total number of temporal and spatial component combinations selected to represent the important patterns after performing TD. “T” represents the number of selected temporal components, and “S” represents the number of selected spatial components used to create the combinations.

#### 3.2.1. Comparison to grand averaging

**Figure 9:**
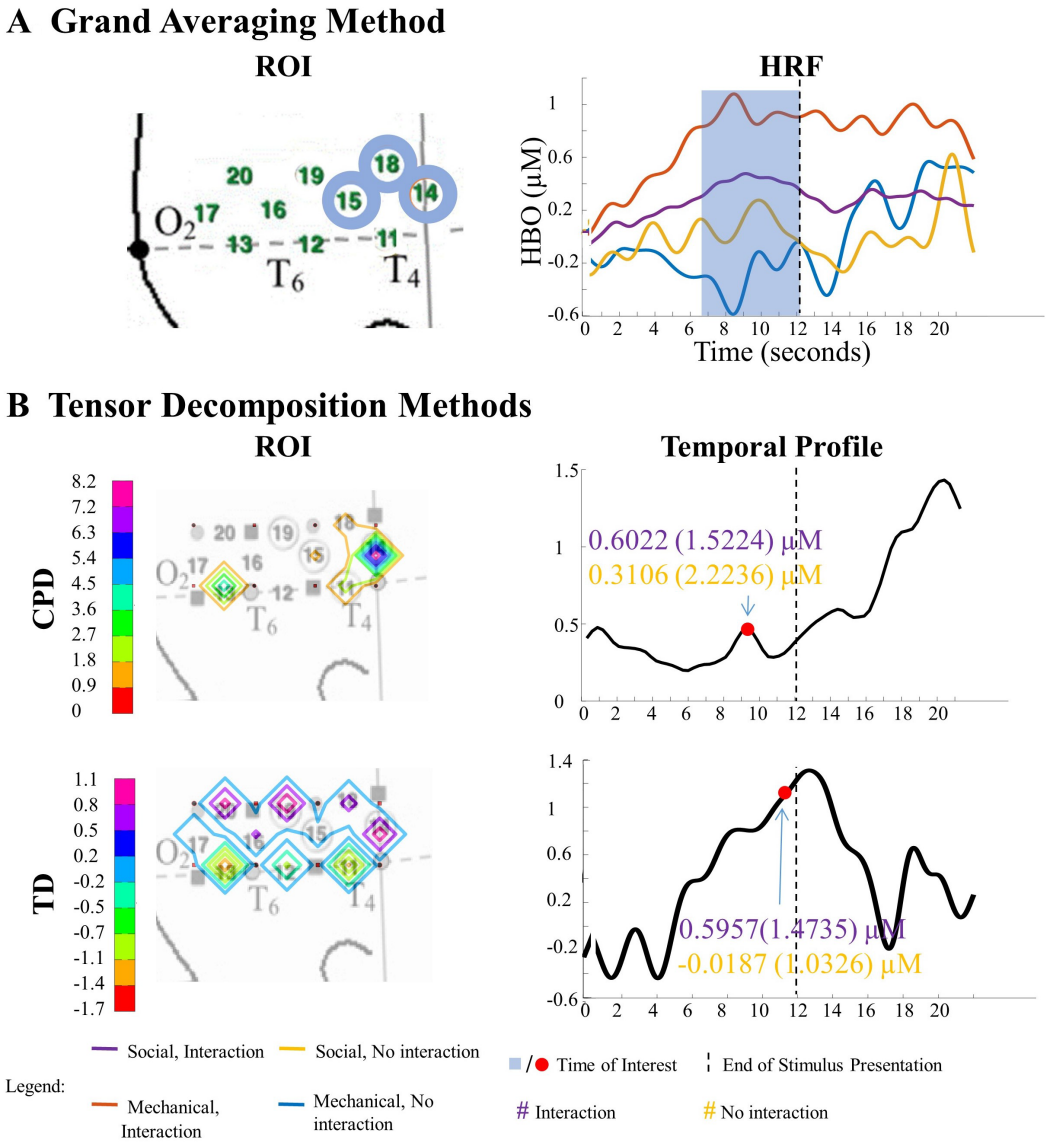
Significant main effect of action sequence in the right hemisphere. **(A)** Grand Averaging Method: The graph displays the significant difference in hemodynamic response between interaction and no interaction for the statistically defined ROI and predefined TOI window within the HRF. **(B)** Tensor Decomposition Method: CPD and TD identified a ROI and TOI.

**Figure 10:**
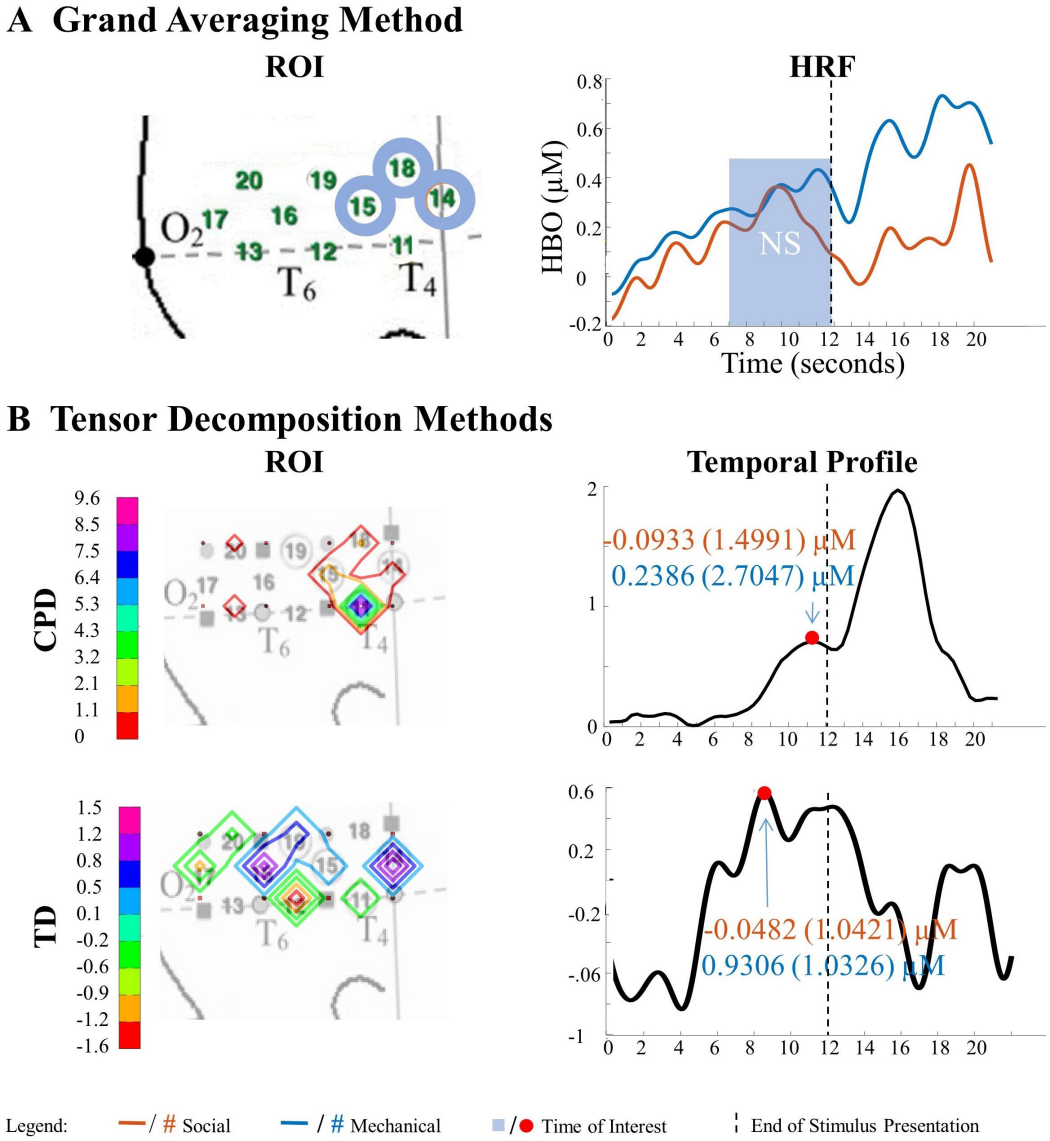
Tensor decomposition method revealed a novel main effect of entity type in right hemisphere. **(A)** Grand Averaging Method: No significant (NS) difference between social and mechanical entities. **(B)** Tensor Decomposition Method: CPD and TD agreed on a novel significant difference and identified a ROI (anterior temporal cortex for CPD and TD) and TOI (second half of stimulus presentation for CPD and TD).

**Figure 11:**
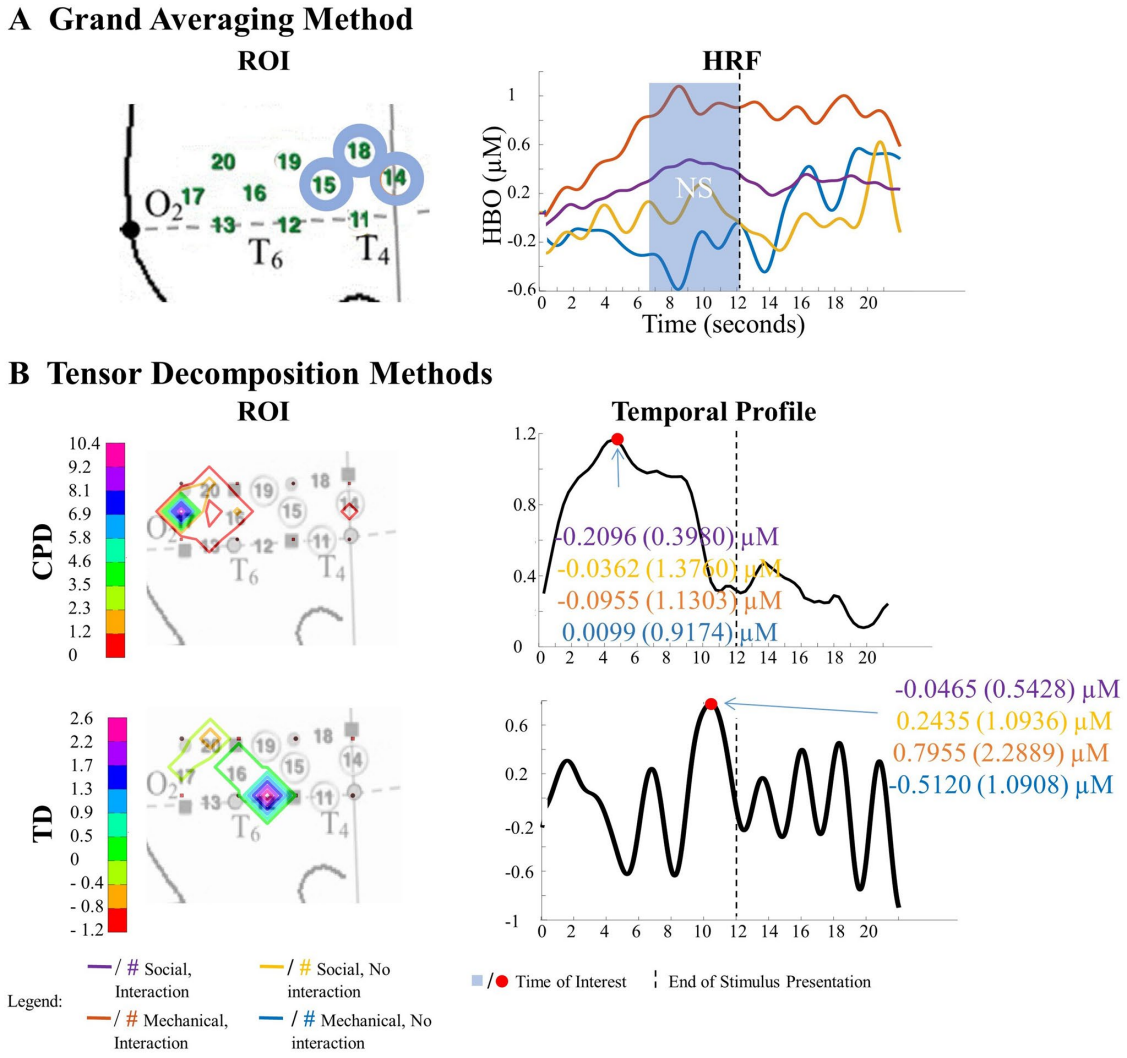
Tensor decomposition method revealed a novel Entity Type × Action Sequence interaction in right hemisphere. **(A)** Grand Averaging Method: No significant (NS) difference across conditions was found for the statistically defined ROI and predefined TOI window within the HRF. **(B)** Tensor Decomposition Method: CPD and TD agreed on a novel significant difference and identified a ROI (temporal-occipital cortex for CPD; middle temporal cortex for TD) and TOI (first half and second half of stimulus presentation for CPD and TD, respectively).

The outcomes of the CPD and TD analyses were compared to the results from the grand averaging method reported by Biondi et al. (2021) in Table 4. Figures 9–11 highlight some more interesting patterns identified in the data, with additional patterns shown in Supplementary Figures S5–S7 and Supplementary Tables S3, S4. The frequency mode information from CPD was not included since no differences were detected across conditions.

**Table 4:** Comparison of the results obtained using the grand averaging method, CPD, and TD on the Social/Mechanical Interactions dataset for both hemispheres.

	Grand averaging	Canonical Polyadic Decomposition (CPD)	Tucker Decomposition (TD)
Main effect of entity type in left hemisphere (Supplementary Figure S5)	NS	Channel 7	Channel 6
Main effect of entity type in Right hemisphere (Figure 10)	NS	Channel 11	Channel 14
Main effect of action sequence in left hemisphere (Supplementary Figure S6)	NS	Channels 6 & 10	Channel 10
Main effect of action sequence in right hemisphere (Figure 9)	channel 14, 15 & 18 (social) channel 15 & 18 (mechanical)	Channel 14	Channels 14, 15, 16, 18, 19, & 20
Entity type × action sequence interaction in left hemisphere Supplementary Figure S7	channel 7	Channel 4	Channel 4
Entity type × action sequence interaction in right hemisphere (Figure 11)	NS	Channel 17	Channel 12 & 16

This table includes patterns for entity type, action sequence, and the interaction between entity type and action sequence. “NS” indicates a nonsignificant effect.

##### 3.2.1.1. Tensor reveals similar results as grand averaging

A statistically significant main effect of action sequence was found in the right hemisphere for the grand averaging method, where there was a greater hemodynamic response to interaction events compared to no interaction events (Figure 9A; Supplementary Table S3 for *F*-values and *p*-values). However, since the ROIs identified for the social and mechanical entity types were not identical, separate one-way ANOVAs were conducted for the social and mechanical stimulus events. For the social entity ROI (channels 14, 15, and 18), there was a significantly greater hemodynamic response to social, interaction event than the social, no interaction event. For the mechanical entity ROI (channels 15 and 18), there was a significantly greater response to the mechanical, interaction event than the mechanical, no interaction event. The CPD and TD analyses revealed similar results as the grand averaging method. CPD revealed differences in the hemodynamic response to interaction events compared to no interaction events in channel 14 during the second half of the stimulus presentation (Figure 9B; Supplementary Table S4 for means and standard deviations of the hemodynamic response calculated from the TOI and ROI indicated by grand averaging, CPD, and TD). The average hemodynamic response within channel 14 showed greater response to interaction than no interaction events. TD identified a main effect of action sequence in channels 14, 15, 16, 18, 19, and 20 during the second half of the stimulus presentation (Figure 9B; Supplementary Table S4). The mean hemodynamic response values obtained at the TOI in the ROI showed a greater response to interaction than no interaction events.

##### 3.2.1.2. Tensor reveals additional results to grand averaging

The grand averaging method did not identify a significant main effect of entity type in the right hemisphere (Figure 10A; Supplementary Table S3). However, the tensor decomposition method revealed a greater response to mechanical entities than social entities during the second half of the stimulus presentation in channel 11 for CPD and channel 14 for TD (Figure 10B; Supplementary Table S4).

The grand averaging method failed to identify a significant Entity Type × Action Sequence interaction in the right hemisphere (Figure 11A; Supplementary Table S3). However, the tensor decomposition method showed that there was an interaction effect (Figure 11B; Supplementary Table S3). CPD found an interaction between entity type and action sequence in channel 17 during the first half of the stimulus presentation. Bayesian analyses were conducted on the mean hemodynamic responses obtained at channel 17 to understand the source of this interaction effect. The analysis showed no difference in response between mechanical entities interacting versus not interacting or between social entities interacting versus not interacting (all BFs < 1, Supplementary Table S4). TD identified an interaction effect in channels 12 and 16 during the second half of the stimulus presentation, with a greater response observed to the interaction event than the no interaction event with mechanical entities (BF = 3.80) and no difference between the interaction and no interaction events with social entities (BF < 1, Supplementary Table S4).

In conclusion, the findings from the Social/Mechanical Interactions dataset support those from the Human Hand/Mechanical Claw dataset, demonstrating that the tensor decomposition method is a more sensitive method of analysis and provides a more comprehensive understanding of the data compared to the grand averaging method.

## 4. General discussion

The current study examined the use of the tensor decomposition method for fNIRS signal analysis. The aim was to determine whether the tensor decomposition method can identify significant hemodynamic response patterns that the traditional grand averaging method missed, as the latter collapses temporal and spatial information, which may also lead to loss of information because the method fails to examine the interactions between modes. Our key findings were that the tensor decomposition method could duplicate the significant results identified with the grand averaging method and identify additional significant hemodynamic response patterns that the grand averaging method failed to detect. The tensor decomposition method was able to identify these significant patterns without having any prior assumptions of the patterns, suggesting that it is a reliable and sensitive technique for fNIRS data analysis.

The ability to detect patterns in hemodynamic responses can significantly increase the accuracy in characterizing these responses. This is demonstrated through the analysis of the Human Hand/Mechanical Claw dataset. The grand averaging method showed a significant interaction between the entity type (human hand or mechanical claw) and action sequence (function or nonfunction), during 8 s to 15 s, in the left anterior/middle temporal cortex (Figure 5A). The two tensor decomposition methods also revealed the same interaction. However, they identified the TOI to be more specifically around 12 s, at which the difference between the human hand and mechanical claw was greatest (Figure 5B). These results show that the tensor decomposition methods are more effective in accurately identifying the specific time and region at which the differences in responses occur compared to the grand averaging method.

One of the key findings was that the tensor decomposition method could reveal brain activation patterns that are not detectable through grand averaging, which can improve our understanding of brain function. Using the Human Hand/Mechanical Claw dataset as an example, the tensor decomposition methods showed a main effect of entity type in anterior/middle temporal cortex channels, meaning that these channels responded differently to the distinction between the human hand and mechanical claw, regardless of the functional relevance of the tool action sequences. This is consistent with previous research that suggests that humans are sensitive to the difference between human and nonhuman/mechanical entities from an early age (Woodward, 2009; Gerson and Woodward, 2012). Furthermore, the study found that the initial response in the anterior temporal cortex is to the difference between human and mechanical entities. However, after viewing the event for an extended period, the response becomes more nuanced, only showing a distinction between human and mechanical entities within the context of functional tool use. This highlights the conditions under which infants are most sensitive to ontological distinctions, which is essential for understanding their cognitive development.

The current results also demonstrate that applying the tensor decomposition method, which can identify patterns of activation not detectable with grand averaging, can significantly improve our conceptual models of brain function. Again, consider the Human Hand/Mechanical Claw dataset. Both tensor decomposition methods, but not grand averaging, identified a main effect of entity type in anterior/middle temporal cortex channels. That is, tensor decomposition identified the channels that responded to the distinction between the human hand and mechanical claw, regardless of whether the tool action sequences were functionally relevant. This outcome is consistent with a large body of literature suggesting that human versus nonhuman/mechanical is an ontological distinction to which humans are sensitive from the early months of life (Woodward, 2009; Gerson and Woodward, 2012).

Furthermore, the study found that the initial response in the anterior temporal cortex was to the difference between human and mechanical entities. However, after viewing the event for an extended period, the response becomes more nuanced, only showing a distinction between human and mechanical entities within the context of functional tool use. This outcome supports the idea that infants are more sensitive to the distinction between human and mechanical entities when viewing dynamic events involving these entities, with the initial response being in the anterior temporal cortex. This sensitivity was greater when the entities were involved in functional tool use and other goal-directed behaviors. This finding is supported by previous research that showed similar results (Gerson and Woodward, 2012; Biondi et al., 2016). This is significant from a theoretical viewpoint as it provides insight into the distinctions between human and mechanical entities that infants are most sensitive to and the conditions that lead to this sensitivity.

When considering tensor decomposition techniques, it is crucial to understand the signal-to-noise ratio of the data being analyzed. TD has been found to perform well on low signal-to-noise ratio data, making it a potential solution to overcome the limitations of CPD (Cong et al., 2013). However, it is important to note that this does not mean that TD is always the better option. For instance, there have been successful applications of CPD on infant EEG datasets, which shows that the choice between the two methods depends on the data being analyzed (Caicedo et al., 2019; De Wel et al., 2019).

When deciding between TD and CPD, familiarity with the fNIRS signal is another key consideration. TD offers more flexibility in selecting components from each mode, allowing for a more accurate examination of combinations of components during decomposition (Kolda and Bader, 2009; Cong et al., 2013; Rabanser et al., 2017). However, this advantage comes at the cost of needing a deeper understanding of the hemodynamic response, which is required to select only the relevant components (Cong et al., 2015). On the other hand, CPD does not require prior knowledge of every mode of the signal, and in the current study was found to be easier to interpret.

Another essential factor to consider when performing tensor decomposition is the type of constraint used. If the data being analyzed is nonnegative, a nonnegativity constraint can be applied as it makes interpretation easier (Cichocki et al., 2009). However, this constraint will not reveal the directionality of the signal. It will only reveal changes in the hemodynamic response, not if they are above or below zero activity. In the present study, a nonnegativity constraint was used for CPD, and an orthogonal constraint was used for TD. The nonnegativity constraint made it easier to interpret the results. However, if the goal is to examine a response below zero (Race et al., 2009), then an orthogonal constraint should be used instead. The choice of constraint can greatly impact the interpretation of results. It is crucial to choose carefully based on the research goals and data characteristics.

One potential direction for future work is to further explore the application of the tensor decomposition method on individual trials rather than just the averaged hemodynamic response. Examining the changes in the signal across trials would provide deeper insights into the changes in the fNIRS signal across the entire experiment and could help shed light on learning effects (Leff et al., 2008). Additionally, by comparing the tensor decomposition method to other methods, such as the GLM, we could gain a deeper understanding of the strengths and limitations of both methods and identify areas where further improvement may be needed. The current study provides a foundation for such future work, and the results could contribute to advancing fNIRS research.

## 5. Conclusion

The current study aimed to investigate the feasibility of the tensor decomposition method in analyzing fNIRS data. Two datasets were utilized in this study, the Human Hand/Mechanical Claw dataset and the Social/Mechanical Interactions dataset, to evaluate the performance of the tensor decomposition method in comparison to the traditional grand averaging method. The results of the study showed that the tensor decomposition method was effective in identifying significant differences across conditions and that it was able to uncover novel hemodynamic response patterns that were not apparent from the grand averaging method. The results from both datasets suggest that the tensor decomposition method is a reliable and sensitive technique for analyzing fNIRS data, as it was found to be more comprehensive and sensitive than the grand averaging method. However, it is important to consider several factors, such as the signal-to-noise ratio of the data and the familiarity with the fNIRS signal, when performing the tensor decomposition method. Overall, the results of the current study support the conclusion that tensor decomposition is a powerful tool in fNIRS analysis, providing a more detailed and nuanced understanding of the data compared to traditional methods. These results have important implications for the study of brain function and could contribute to the progression of our understanding in this field.

## Data availability statement

The data analyzed in this study is subject to the following licenses/restrictions: The datasets analyzed for this study will be made available upon request. Requests to access these datasets should be directed to wilcoxt@fau.edu.

## Ethics statement

The studies involving human participants were reviewed and approved by Texas A&M University and Florida Atlantic University. Written informed consent to participate in this study was provided by the participants’ legal guardian/next of kin.

## Author contributions

BG and TW conceptualized the experiment and provided feedback. TW provided the datasets. MH and JC performed the background research and analysis. JC wrote the first draft. MH and TW wrote sections. All authors contributed to the article and approved the submitted version.

## Funding

Collection of the infant fNIRS data was partly supported by the National Institutes of Health (R01-HD057999) to TW. The data analytics aspect of this study was partly supported by the National Science Foundation (1936586 and 1942669) to BG, PI.

## Conflict of interest

The authors declare that the research was conducted without any commercial or financial relationships that could be construed as a potential conflict of interest.

## Publisher’s note

All claims expressed in this article are solely those of the authors and do not necessarily represent those of their affiliated organizations, or those of the publisher, the editors and the reviewers. Any product that may be evaluated in this article, or claim that may be made by its manufacturer, is not guaranteed or endorsed by the publisher.
