# Supplementary material for: Exploring the feasibility of tensor decomposition for analysis of fNIRS signals: a comparative study with grand averaging method
Source: Front Neurosci. 2023 Aug 10;17:1180293. doi: 10.3389/fnins.2023.1180293 (PMC10448703; doi:10.3389/fnins.2023.1180293)
Supplement: Supplementary file 1 [file Data_Sheet_1.docx]

Supplementary Material

# Supplementary Dataset and Data Preprocessing

The imaging equipment was a TechEn CW7 (TechEn Inc. MA, USA). Participants' were fitted with a custom-made headgear containing 8 fiber optic cables that delivered near-infrared light to the participates scalp (sources) and 8 optic cables that detected the diffusely reflected light at the scalp detectors (detectors). Configuration of the sources and detectors within the headgear, location of the corresponding channels, and placement of the headgear on the infants’ head in relation to the 10-20 International EEG system are illustrated in Figure S1. The fibers were connected to a control box that produced light at wavelengths of 690, which is more sensitive to deoxygenated blook (HBR), and 830, which is more sensitive to oxygenated blood (HBO). Infants’ head sizes ranged from 41.5 cm to 49 cm. The difference between the amount of skull covered from the left and right parts of the headgear was 1.69 cm when comparing from the smallest to largest head circumference, which is less than the distance between the source and detector.

The sampling frequency of the raw signals were collected at 50 Hz (*N*= 70, for the Human Hand/Mechanical Claw data set; *n =* 30, for the Social/Mechanical Interactions data set) and 25 Hz (*n*= 6, for the Social/Mechanical Interactions data set). The fNIRS signal collected at 25 Hz for the Social/Mechanical Interactions data set was upsampled with spline interpolation to match the number of data points collected with the 50 Hz sampling frequency. Preprocessing of neuroimaging data was done with HOMER2 (Huppert et al., 2009), an open software that was programmed with MATLAB 2016a (Matworks, Natick, MA). The quality of each channel was checked and pruned (dRange = 1e03 [for the Human Hand/Mechanical Claw data set] or 1e04 [for the Social/Mechanical Interactions data set] to 1e07, SNRthresh = 2, SDrange = 0.0 to 45.0, reset = 0). Intensity of the photons was converted to optical density units. Systemic noise and motion artifacts were detected and removed using principal component analysis ([PCA] nSV = 0.80). Any additional motion artifacts that were not corrected for with PCA were identified (tMotion = 0.5, tMask = 1.0, STDEVthresh = 50.0, AMPthresh = 2.0). Trials that had motion artifacts occur between 2 s before the presentation of the stimulus and the end of stimulus presentation (15 s for the Human Hand/Mechanical Claw data set and 12 s for the Social/Mechanical Interactions data set) were excluded from the analysis. The signal was low-pass filtered at 0.1 Hz, for the Human Hand/Mechanical Claw data set, or bandpass filtered from 0.1 to 0.5 Hz, for the Social/Mechanical Interactions data set, using a third-order Butterworth filter. The optical density of the signal was converted to relative hemoglobin concentrations using the modified Beer-Lambert Law (ppf = 6.0 6.0). Stimulus marks that fell within our selection criteria were excluded (Stim_Include_UserDefVar = 8.0 15 for the Human Hand/Mechanical Claw data set and 7.0 12.0 for the Social/Mechanical Interactions data set). Hemodynamic response functions included the 2 s before the onset of the stimulus through 25 s, for the Human Hand/Mechanical Claw data set, or 22 s, for the Social/Mechanical Interactions data set, after the onset of the stimulus. Changes in hemodynamic responses were measured by setting the baseline (-2 s to 0 s) proceeding the onset of the stimuli to zero and the hemodynamic responses were assessed from zero.

# Supplementary Grand Averaging Method

We focused on the two main modes over which fNIRS data were averaged in the previously published datasets: temporal and spatial. In both datasets, the grand averaging method was applied. The process began with the HRFs from both datasets being averaged over a predefined TOI window to create a single temporal mean value for each channel. The predefined TOI window was 8 s to 15 s for the Human Hand/Mechanical Claw dataset (Biondi et al., 2016) and 7 s to 12 s for the Social/Mechanical Interactions dataset (Biondi et al., 2021).

For the two datasets, slightly different approaches were taken, to create ROIs to test the prediction that condition-unique response patterns would be obtained. For the Human Hand/Mechanical Claw dataset (Biondi et al., 2016), temporal mean values obtained at each channel were subjected to a 2 (entity type) × 2 (action sequence) ANOVA for each hemisphere separately (α = 0.05 was used for all analyses reported). Spatially contiguous channels that identified a significant main effect of entity type, main effect of action sequence, or Entity Type × Action Sequence interaction were grouped by the main effect or interaction and averaged to form spatial mean values for the ROI. A 2 (entity type) × 2 (action sequence) ANOVA was performed on the spatial mean values to confirm the channel level analysis. Paired comparisons were used to understand the directionality of the significant Entity Type × Action Sequence interactions.

For the Social/Mechanical Interactions dataset (Biondi et al., 2021), the single temporal mean value obtained at each channel, for each entity type and action sequence, was compared to 0 with a one-tailed t-test (α = 0.05). One-tailed t-tests were used because oxygenated hemoglobin responses greater than 0 were predicted. To create ROIs, spatially contiguous channels that showed a significant oxygenated hemoglobin response were grouped into ROIs by entity type and t-tests were performed to assess the extent to which the mean oxygenated hemoglobin responses obtained in the ROI differed by test event (i.e., social versus mechanical).

# Supplementary Proposed Tensor Decomposition Method

## Tensor Construction

In preparation for the tensor decomposition method, a four-way tensor with temporal × spectral × spatial × subject modes (X $\in I_{t} \times I_{f} \times I_{c} \times I_{s}$) was constructed for each dataset. $I_{t}$indicates the number of temporal samples, $I_{f}$ the number of spectral samples, $I_{c}$the number of channels, and $I_{s}$the number of subjects. First, we created time-frequency representations of each averaged trial. We used Short-time Fourier transform based on a 3 s Kaiser window with a beta of 5 and 90% overlapping. Next, the magnitude calculated from 0 Hz - 1 Hz of the Short-time Fourier transform of each channel from every subject was arranged into the tensor structure. The range 0 Hz -1 Hz was used to perform the non-negativity constraint.

In preparation for the other tensor decomposition technique, a three-way tensor with temporal × spatial × subject modes (Y $\in I_{t} \times I_{c} \times I_{s}$) was constructed for each dataset. $I_{t}$indicates the number of temporal samples, $I_{c}$ the number of channels, and $I_{s}$ the number of subjects.

## Tensor Decomposition

Generally, CPD decomposes an original tensor X with *N* number of modes, $u,$ into *R* number of components $u_{r}^{(1:N)}$plus error *E* (Carroll & Chang, 1970; Cichocki et al., 2015; Kolda & Bader, 2009; Rabanser et al., 2017; see Equation 1). The identity matrix $I$ has ones along the diagonal of the matrix and zeros for the rest. Component matrix, $U,$ represents all the components from each mode number, $N$. The components from each mode $u_{r}^{(1:N)}$ represent the main underlying patterns, and error *E* represents the background outlier information (Cong et al., 2015). Hence, the components (e.g., $u_{r}^{(1:N)}$) for the approximate tensor X will provide the main information representing the underlying dynamics of the system generated from the original tensor X.

X = $\sum_{r=1}^{R} u_{r}^{(1)}{。u}_{r}^{(2)}。\ldots{。u}_{r}^{\left( N \right)}$+ E

*≈* $I\times_{1}U^{(1)}\times_{2}U^{(2)}\times\ldots\times_{N}U^{(N)}$ (1)

Different symbols were used in the equations to avoid confusion between CPD and TD. In general, TD decomposes the original tensor, Y, with *N* number of modes into a core tensor ($g$), $R_{N}$ number of components from each mode ${(a}^{(N)})$, and error *E* (Cong et al., 2015; Kolda & Bader, 2009; Rabanser et al., 2017; Tucker, 1966; see Equation 2). The core tensor consists of the product of the components from each mode, $G\in R^{R_{1}\times\ldots\times R_{N}}$, thus the core tensor, $G$, represent the main underlying patterns by representing how the $R_{N}$*^th^* components from each mode connect to each other (Zubair & Wang, 2013). Component matrix, $A^{(N)},$ represents all the components from each mode number $N$, e.g., $A^{(1)}\in R^{I_{1}\times R_{1}}$ (Phan & Cichocki, 2010). Error, *E*, represents the background outlier information. Hence, the components in the core tensor, $G,$ will provide the main information representing the underlying dynamics of the system generated from the original tensor, Y.

Y = $\sum_{r_{1}=1}^{R_{1}} \sum_{r_{2}=1}^{R_{2}} \ldots\sum_{r_{N}=1}^{R_{N}} g_{r_{1}r_{2}\ldots r_{N}}a_{r_{1}}^{(1)}。a_{r_{2}}^{(2)}。\ldots{。a}_{r_{N}}^{(N)}$+E

*≈* $G\times_{1}A^{(1)}\times_{2}A^{(2)}\times\ldots\times_{N}A^{(N)}$ (2)

For CPD, the number of components, $R$, extracted should have a reconstruction error rate below 10%. For TD, the same method was used to estimate the number of components, $R^{R_{t} \times R_{c} \times I_{s}}$, that should have been extracted.

## Determination of TOI and ROI

Following the exclusion of irrelevant components, ANOVA was applied. The statistically significant temporal ($u_{r}^{(t)}$for CPD and $a_{r_{t}}^{(t)}$ for TD) and spatial ($u_{r}^{(c)}$ for CPD and $a_{r_{c}}^{(c)}$ for TD) (and spectral [$u_{r}^{(f)}$ for CPD]) components were summed together in the according significant effects and hemisphere to represent the temporal profile and ROI (and spectral profile for CPD), respectively, to identify response differences across conditions. $Z$ lists all the statistically significant components from CPD. $Z_{t}$ and $Z_{c}$ lists all the significant temporal and spatial components from TD, respectively. For example, if the 1^st^, 3^rd^, and 5^th^ temporal components demonstrated a significant main effect of entity type, then $Z_{t}$ = {1,3,5}. Equations 3-5 demonstrate how the temporal profile, ROI, and spectral profile, respectively, were constructed for CPD and Equations 6 and 7 for TD.

temporal profile for CPD =$\sum_{r\in Z} u_{r}^{(t)}$ (3)

ROI for CPD = $\sum_{r\in Z} u_{r}^{(c)}$ (4)

spectral profile for CPD = $\sum_{r\in Z} u_{r}^{(f)}$ (5)

temporal profile for TD = $\sum_{r\in Z_{t}} a_{r_{t}}^{(t)}$ (6)

ROI for TD = $\sum_{r\in Z_{c}} a_{r_{c}}^{(c)}$ (7)

# Supplementary Figures


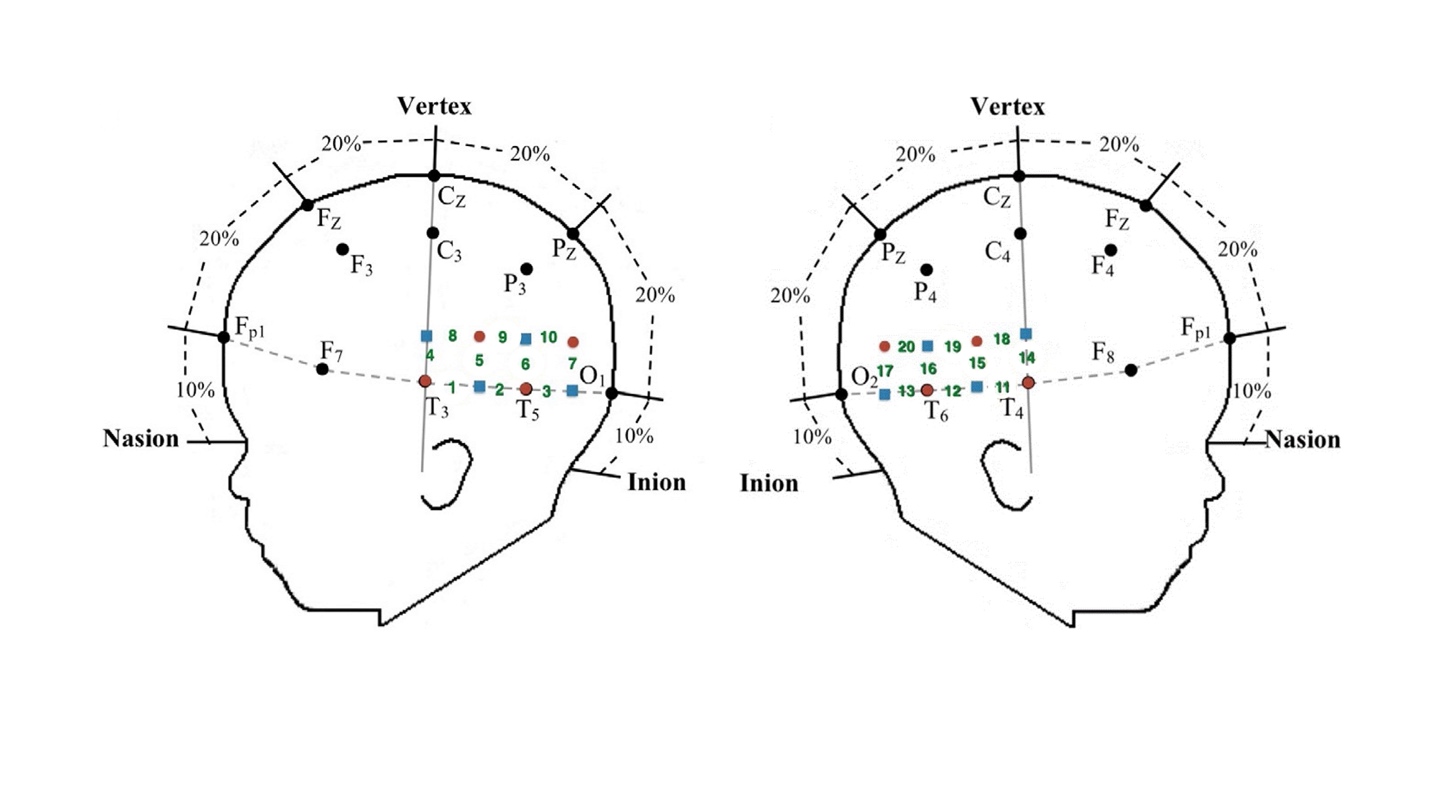


**Figure S1.** Headgear configuration and placement. The headgear consisted of two pads, placed bilaterally over temporal and temporal-occipital cortex, as previously described by Biondi et al., in 2016 and 2021. Four sources (red circles) and four detectors (blue squares) were embedded in each of the two pads (for a total of 8 sources and 8 detectors). Source-detector pairings created 10 channels in each hemisphere (for a total of 20 channels). The numbers are the channels. The left and right pads were anchored at T3 and T4 (with O1 and O2 as secondary anchors), respectively, of the 10-20 International EEG system. Source-detector distances were 2 cm.


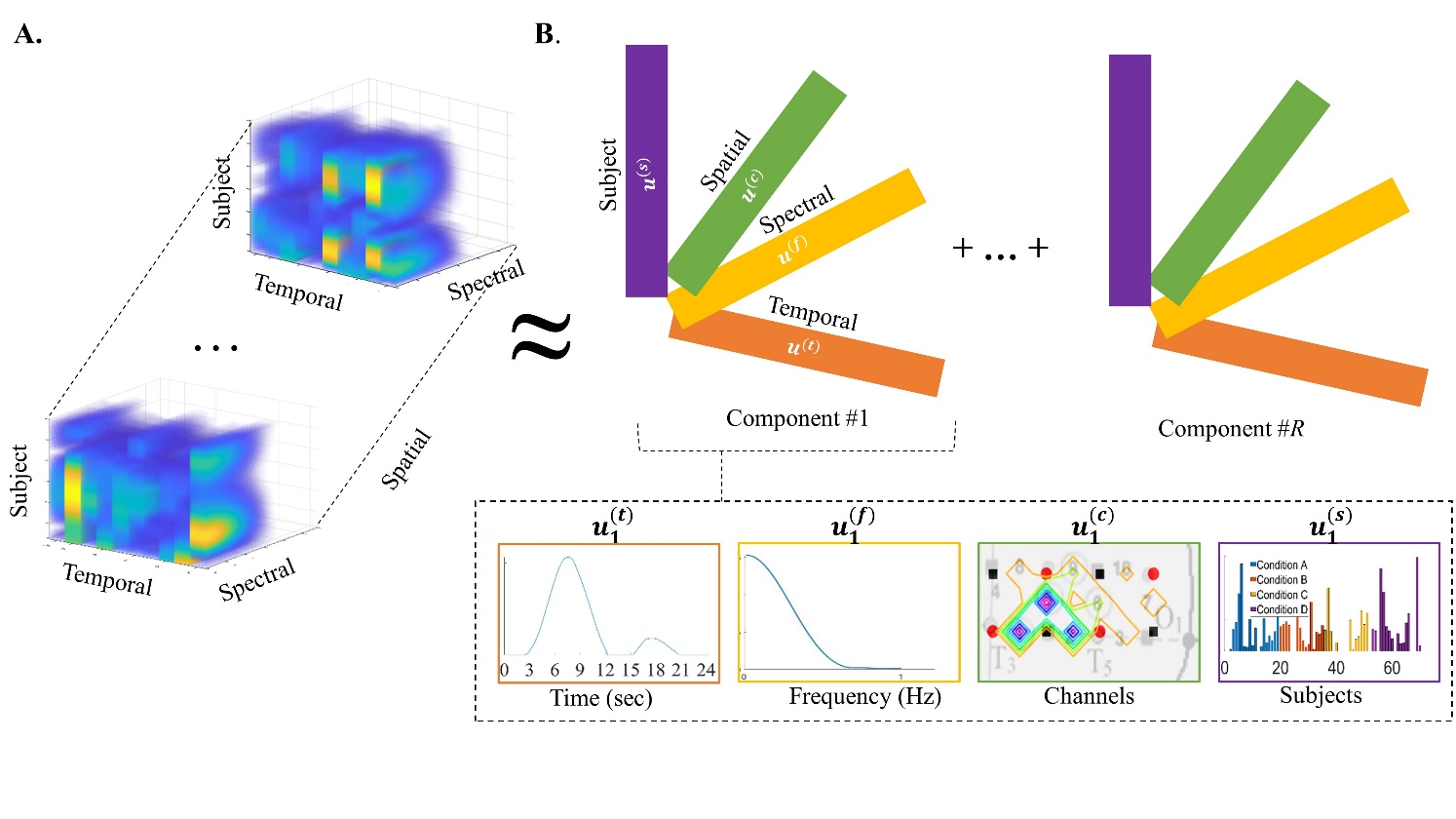


**Figure S2.** Tensor construction and canonical polyadic decomposition (CPD) (A) Illustrates the construction of a four-way tensor with temporal, spectral, spatial, and subject modes*.* (B) Illustration of the temporal, spectral, spatial, and subject components from CPD, including an example of a component from each mode.


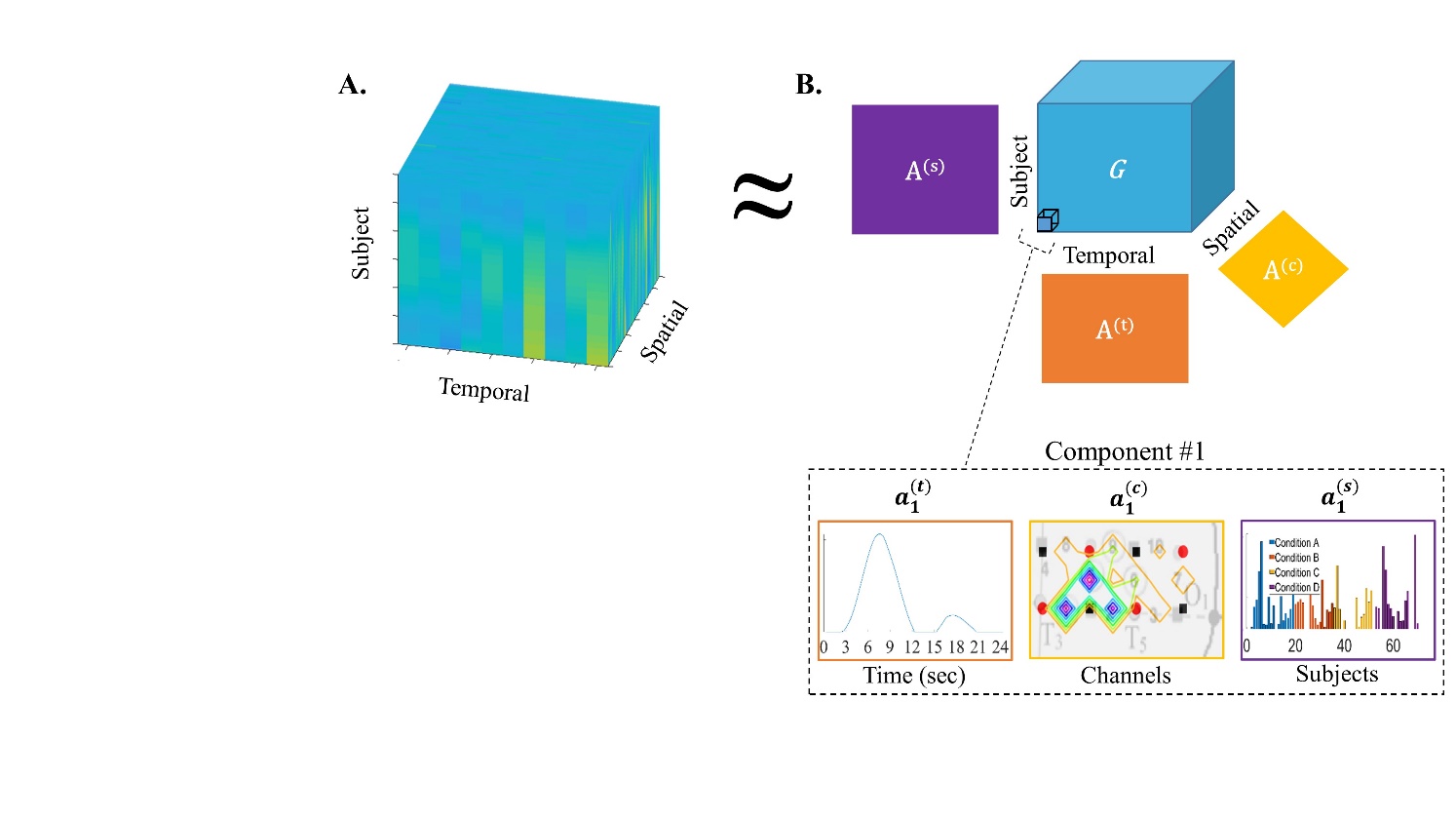


**Figure S3.** Tensor construction and Tucker Decomposition (TD) (A) Illustrates the construction of a three-way tensor with temporal, spatial, and subject modes*.* (B) Illustration of the temporal, spatial, and subject components from TD, including an example of a component from each mode.


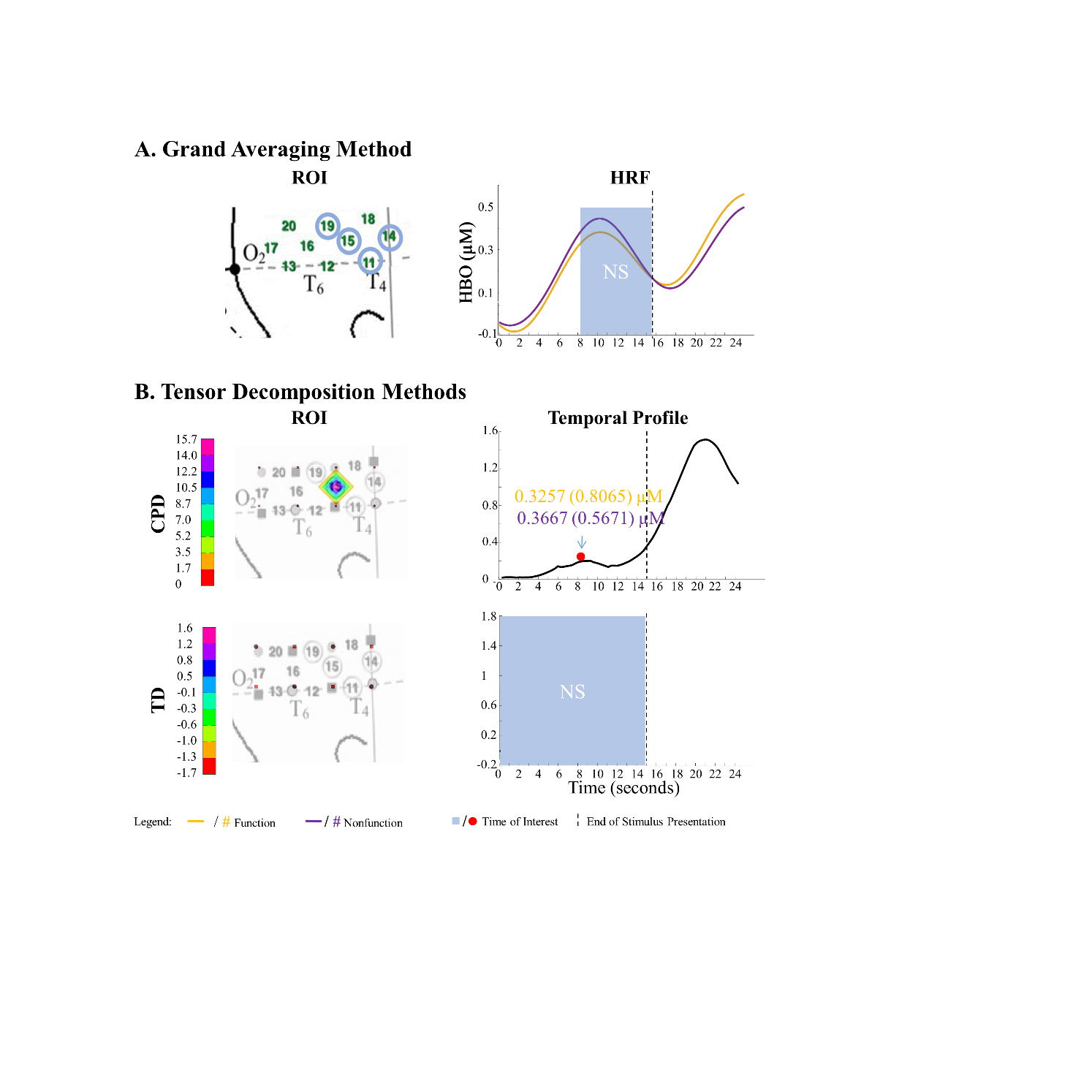


**Figure S4.** CPD revealed a novel main effect of action sequence in right hemisphere. (A) Grand Averaging Method: No significant (NS) difference between function and nonfunction events was found for the statistically defined ROI and predefined TOI window within the HRF. (B) Tensor Decomposition Method: CPD, not TD, revealed a novel significant difference and identified a ROI (middle temporal cortex) and TOI (second half of stimulus presentation).


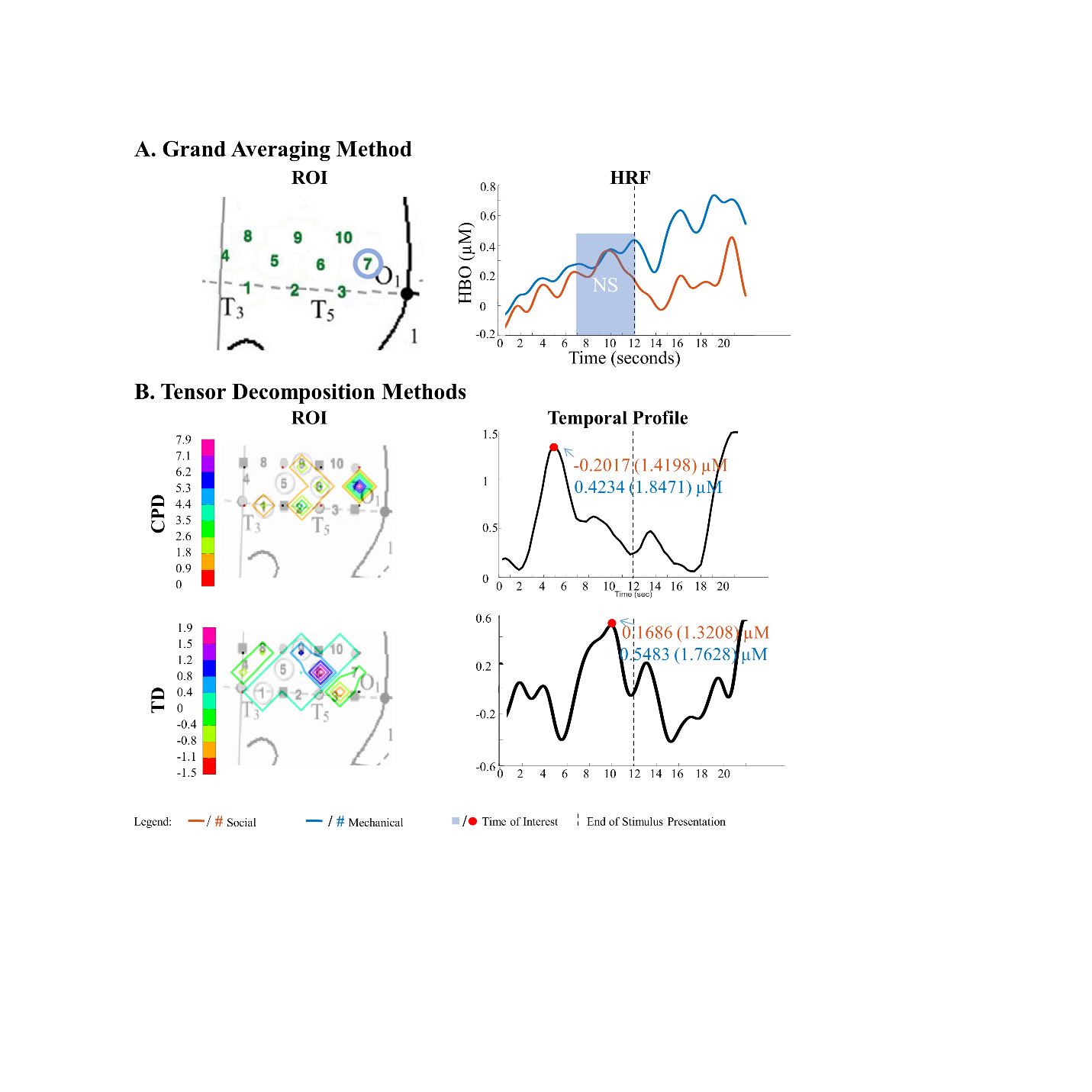


**Figure S5**. Tensor decomposition method revealed a novel main effect of entity type in left hemisphere. (A) Grand Averaging Method: No significant (NS) difference between social and mechanical entities was found for the statistically defined ROI and predefined TOI window within the HRF. (B) Tensor Decomposition Method: CPD and TD agreed on a novel significant difference and identified a ROI (occipital cortex for CPD; posterior temporal cortex for TD) and TOI (first half and second half of stimulus presentation for CPD and TD, respectively).


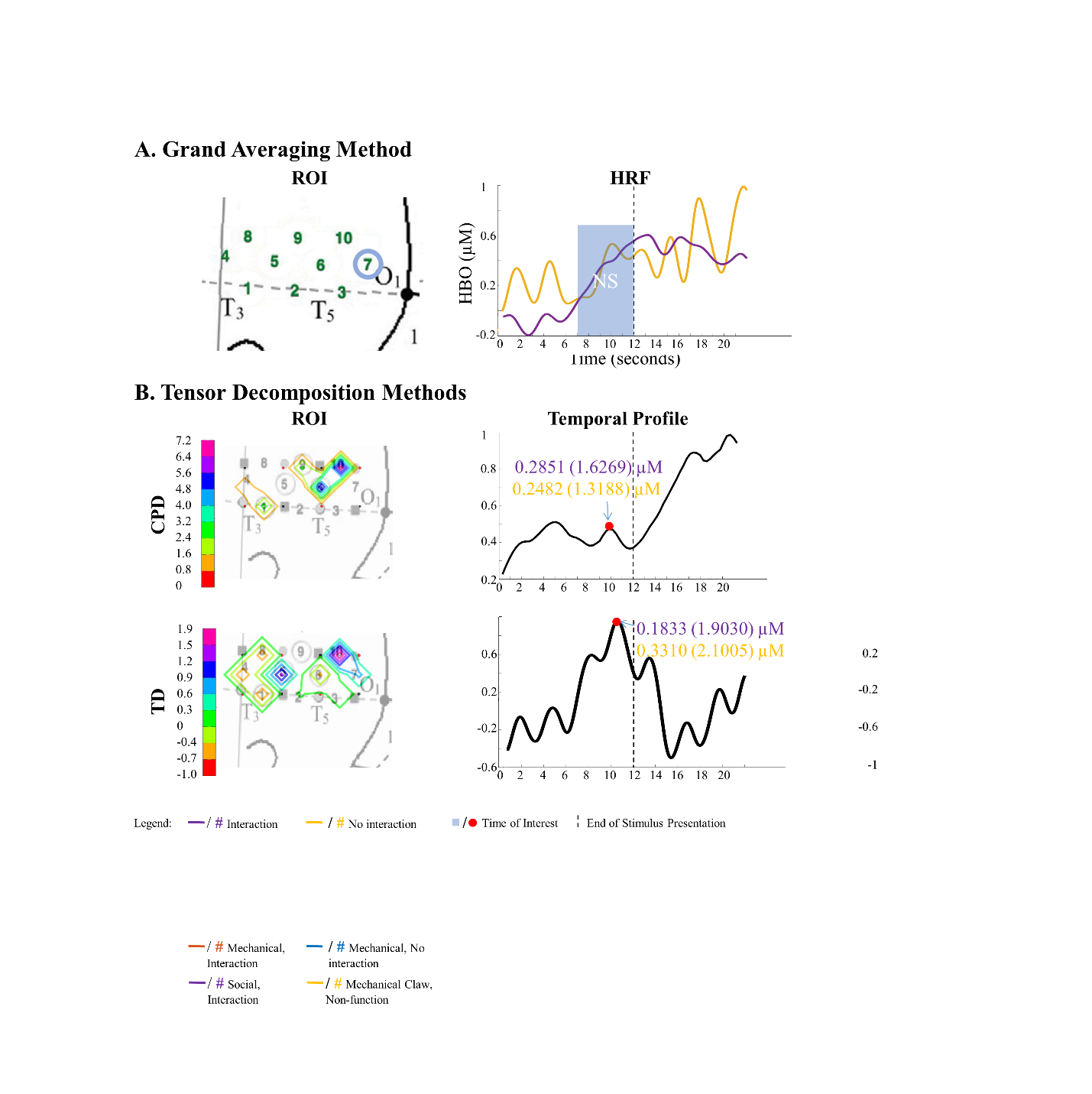


**Figure S6.** Tensor decomposition method revealed a novel main effect of action sequence in left hemisphere. (A) Grand Averaging Method: No significant (NS) difference between interaction and no interaction events was found for the statistically defined ROI and predefined TOI window within the HRF. (B) Tensor Decomposition Method: CPD and TD agreed on a novel significant difference and identified a ROI (temporal-occipital cortex for CPD and TD) and TOI (second half of stimulus presentation for CPD and TD).


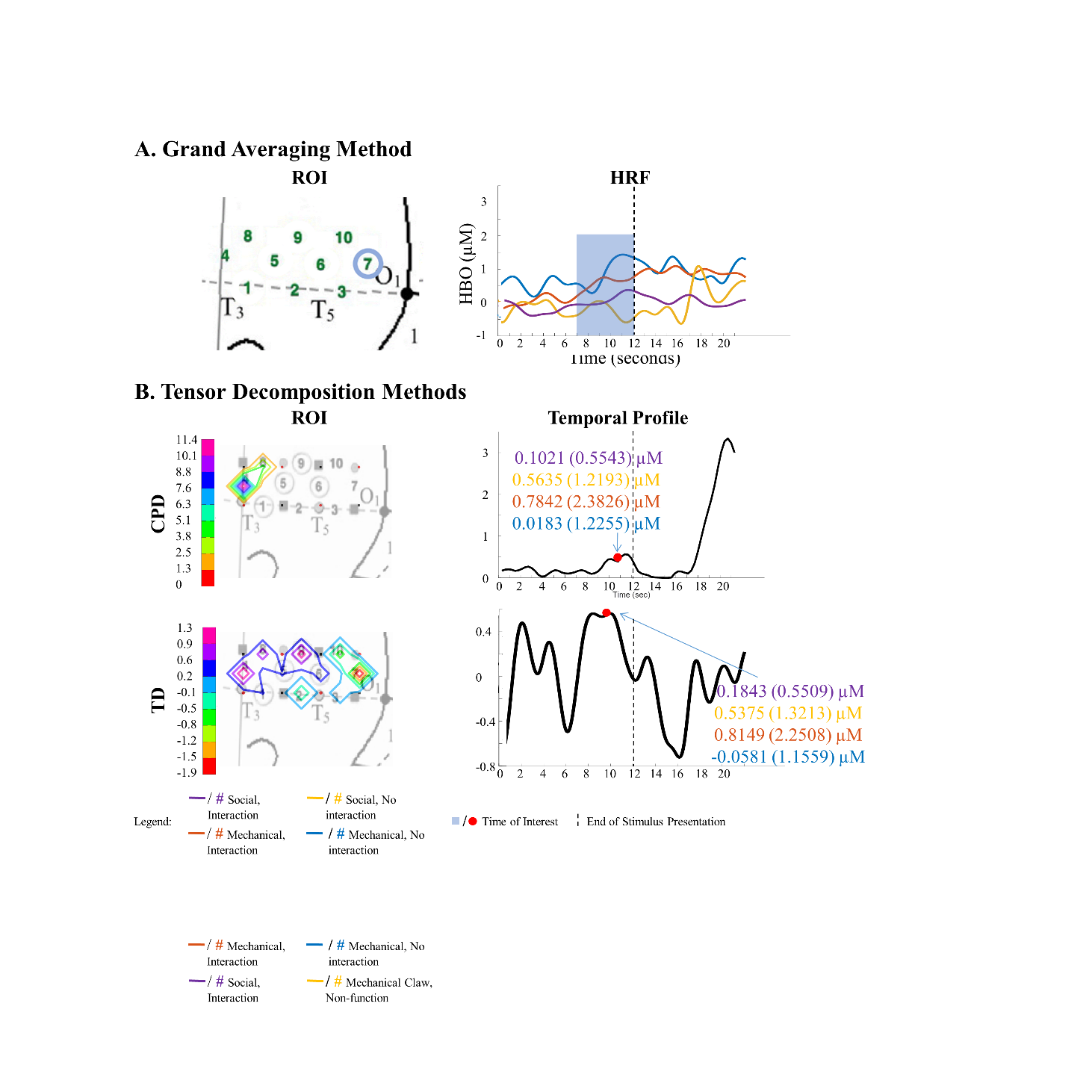


**Figure S7.** Significant Entity Type × Action Sequence interaction in left hemisphere. (A) Grand Averaging Method: The graph displays the significant difference in hemodynamic response across conditions for the statistically defined ROI and predefined TOI window within the HRF. (B) Tensor Decomposition Method: CPD and TD identified a ROI and TOI.

**Table S1.** F-values and p-values obtained from ANOVA when using the grand averaging method, CPD, and TD on the Human Hand/Mechanical Claw dataset. This table includes patterns for entity type, action sequence, and the interaction between entity type and action sequence. “NS” indicates a nonsignificant effect.

|  | Grand Averaging | Canonical Polyadic Decomposition (CPD) | Tucker Decomposition (TD) |
| --- | --- | --- | --- |
| Main Effect of Entity Type in Left Hemisphere  (Figure 7) | - NS - *F*(1,66) = 2.253 - *p* > 0.05 | - *F*(1,66) < 4.088 - *p <* 0.05 | - *F*(1,66) < 4.137 - *p <* 0.05 |
| Main Effect of Entity Type in Right Hemisphere  (Figure 6) | - *F*(1,65) = 14.502 - *p <* 0.001 | - *F*(1,65) < 4.052 - *p <* 0.05 | - *F*(1,65) < 4.431 - *p <* 0.05 |
| Main Effect of Action Sequence in Left Hemisphere | - NS - *F*(1,66) = 2.209 - *p* > 0.05 | - NS - *F*(1,66) < 4.039 - *p <* 0.05 | - *F*(1,66) < 4.081 - *p <* 0.05 |
| Main Effect of Action Sequence in Right Hemisphere (Figure S4) | - NS - *F*(1,65) = 0.111 - *p* > 0.05 | - *F*(1,65) = 4.041 - *p <* 0.05 | - NS - *F*(1,65) < 3.399 - *p >* 0.05 |
| Entity Type × Action Sequence Interaction in Left Hemisphere (Figure 5) | - *F*(1,66) = 15.112 - *p <* 0.001 | - *F*(1,66) < 4.171 - *p <* 0.05 | - *F*(1,66) < 4.839 - *p <* 0.05 |
| Entity Type × Action Sequence Interaction in Right Hemisphere (Figure 8) | - NS - *F*(1,65) = 1.351 - *p* > 0.05 | - *F*(1,65) = 4.040 - *p <* 0.05 | - *F*(1,65) < 4.221 - *p >* 0.05 |

**Table S2.** Means and standard deviation of the hemodynamic response (in μM) obtained from using the grand averaging method, CPD, and TD on the Human Hand/Mechanical Claw dataset. For grand averaging, values were averaged over the TOI (8 s – 15 s) and ROI. For CPD and TD, the mean hemodynamic response and standard deviation were calculated by averaging 1 s before and after the identified TOI within the ROI. This table includes patterns for entity type, action sequence, and the interaction between entity type and action sequence. “NS” indicates a nonsignificant effect.

|  | Grand Averaging | Canonical Polyadic Decomposition (CPD) | Tucker Decomposition (TD) |
| --- | --- | --- | --- |
| Main Effect of Entity Type in Left Hemisphere  (Figure 7) | - NS | - human hand: *M* = 0.0850, *SD* = 0.4647) - mechanical claw: *M* = -0.2304, *SD* = 0.5737 | - human hand: *M* = 0.1916, *SD* = 0.5972) - mechanical claw: *M* = -0.1957, *SD* = 0.5845 |
| Main Effect of Entity Type in Right Hemisphere  (Figure 6) | - human hand: *M* = 0.5591, *SD* = 0.4407 - mechanical claw: *M* = 0.1371, *SD* = 0.6830 | - human hand: *M* = 0.0944, *SD* = 0.4401 - mechanical claw: *M* = -0.0737, *SD* = 0.6996 | - human hand: *M* = 0.5273, *SD* = 0.6440 - mechanical claw: *M* = 0.1287, *SD* = 0.9308 |
| Main Effect of Action Sequence in Left Hemisphere | - NS | - NS | - NS |
| Main Effect of Action Sequence in Right Hemisphere (Figure S4) | - NS | - function: *M* = 0.3257, *SD* = 0.8065 - nonfunction: *M* = 0.3667, *SD* = 0.5671 | - NS |
| Entity Type × Action Sequence Interaction in Left Hemisphere (Figure 5) | - human hand, function: *M* = 0.5467, *SD* = 0.6464 - human hand, nonfunction: *M* = 0.1547, *SD* = 0.4534 - mechanical claw, function: *M* = -0.0977, *SD* = 0.4705 - mechanical claw, nonfunction: *M* = 0.4147, *SD* = 0.5381 | - human hand, function: *M* = 0.8153, *SD* = 0.8845 - human hand, nonfunction: *M* = 0.1631, *SD* = 0.6338 - mechanical claw, function: *M* = 0.0051, *SD* = 0.6842 - mechanical claw, nonfunction: *M* = 0.4142, *SD* = 0.0691 | - human hand, function: *M* = 0.4717, *SD* = 0.6745 - human hand, nonfunction: *M* = 0.1168, *SD* = 0.4009 - mechanical claw, function: *M* = -0.1401, *SD* = 0.5257 - mechanical claw, nonfunction: *M* = 0.2809, *SD* = 0.5257 |
| Entity Type × Action Sequence Interaction in Right Hemisphere (Figure 8) | - NS | - human hand, function: *M* = 0.4020, *SD* = 0.5786 - human hand, nonfunction event: *M* = 0.1125, *SD* = 0.4149 - mechanical claw during function: *M* = -0.1671, *SD* = 0.8354 - mechanical claw, nonfunction: *M* = 0.1600, *SD* = 0.6833 | - human hand, function: *M* = 0.5437, *SD* = 0.6194 - human hand, nonfunction: *M* = 0.3869, *SD* = 0.4856 - mechanical claw, function: *M* = 0.0988, *SD* = 0.8531 - mechanical claw, function: *M* = 0.2889, *SD* = 0.7981 |

**Table S3.** F-values and p-values obtained from ANOVA when using the grand averaging method, CPD, and TD on the Social/Mechanical Interactions dataset. This table includes patterns for entity type, action sequence, and the interaction between entity type and action sequence. “NS” indicates a nonsignificant effect.

|  | Grand Averaging | Canonical Polyadic Decomposition (CPD) | Tucker Decomposition (TD) |
| --- | --- | --- | --- |
| Main Effect of Entity Type in Left Hemisphere (Figure S5) | - NS | - *F*(1,66) < 4.131 - *p <* 0.05 | - *F*(1,66) < 0 - *p <* 0.05 |
| Main Effect of Entity Type in Right Hemisphere (Figure 10) | - NS | - *F*(1,66) < 4.264 - *p <* 0.05 | - *F*(1,66) < 0 - *p <* 0.05 |
| Main Effect of Action Sequence in Left Hemisphere (Figure S6) | - NS | - *F*(1,66) < 4.153 - *p <* 0.05 | - *F*(1,66) < 0 - *p <* 0.05 |
| Main Effect of Action Sequence in Right Hemisphere (Figure 9) | - social: *t*(17) = 4.57, *p <* 0.001 - mechanical: *t*(15) = 3.21, *p =* 0.003 | - *F*(1,66) < 4.153 - *p <* 0.05 | - *F*(1,66) < 0 - *p <* 0.05 |
| Entity Type × Action Sequence Interaction in Left Hemisphere (Figure S7) | - *t*(29) = -2.70, *p =* 0.011 | - *F*(1,66) < 4.142 - *p <* 0.05 | - *F*(1,66) < 0 - *p <* 0.05 |
| Entity Type × Action Sequence Interaction in Right Hemisphere (Figure 11) | - NS | - *F*(1,66) < 4.238 - *p <* 0.05 | - *F*(1,66) < 0 - *p <* 0.05 |

**Table S4.** Means and standard deviation of the hemodynamic response (in μM) obtained from using the grand averaging method, CPD, and TD on the Social/Mechanical Interactions dataset. For grand averaging, values were averaged over the TOI (7 s – 12 s) and ROI. For CPD and TD, the mean hemodynamic response and standard deviation were calculated by averaging 1 s before and after the identified TOI within the ROI. This table includes patterns for entity type, action sequence, and the interaction between entity type and action sequence. “NS” indicates a nonsignificant effect.

|  | Grand Averaging | Canonical Polyadic Decomposition (CPD) | Tucker Decomposition (TD) |
| --- | --- | --- | --- |
| Main Effect of Entity Type in Left Hemisphere (Figure S5) | - NS | - social*: M* = -0.2017, *SD* = 1.4198 - mechanical: *M* = 0.4234, *SD* = 1.8471 | - social*: M* = 0.1686, *SD* = 1.3208 - mechanical: *M* = 0.5483, *SD* = 1.7628 |
| Main Effect of Entity Type in Right Hemisphere (Figure 10) | - NS | - social*: M* = -0.0933, *SD* = 1.4991 - mechanical: *M* = 0.2386, *SD* = 2.7047 | - social: *M* = -0.0482, *SD* = 1.0326 - mechanical: *M* = 0.9306, *SD* = 1.0326 |
| Main Effect of Action Sequence in Left Hemisphere (Figure S6) | - NS | - interaction: *M* = 0.2851, *SD* = 1.6269 - no interaction: *M* = 0.2482, *SD* = 1.3188 | - interaction: *M* = 0.1833, *SD* = 1.9030 - no interaction: *M* = 0.3310, *SD* = 2.1005 |
| Main Effect of Action Sequence in Right Hemisphere (Figure 9) | - social, interaction: *M* = 0.252, *SD* = 0.301 - social, no interaction: *M* = 0.006, *SD* = 0.674 - mechanical, interaction: *M* = 0.684, *SD* = 1.246 - mechanical, no interaction: *M* = -0.032, *SD* = 1.081 | - interaction: *M* = 0.6022, *SD* = 1.5224 - no interaction: *M* = 0.3106, *SD* = 2.2236 | - interaction: *M* = 0.5957, *SD* = 1.4735 - no interaction: *M* = -0.0187, *SD* = 1.0326 |
| Entity Type × Action Sequence Interaction in Left Hemisphere (Figure S7) | - mechanical, no interaction: *M* = 0.764, *SD* = 1.158 - mechanical, interaction: *M* = 0.522, *SD* = 2.268 - social, no interaction: *M* = -0.075, *SD* = 1.384 - social, interaction: *M* = -0.009, *SD* = 0.381 | - social, interaction: *M* = 0.1021, *SD* = 0.5543 - social, no interaction: *M* = 0.5635, *SD* = 1.2193 - mechanical, interaction: *M* = 0.7842, *SD* = 2.3826 - mechanical, no interaction: *M* = 0.0183, *SD* = 1.2255 | - social, interaction: *M* = 0.1843, *SD* = 0.5509 - social, no interaction: *M* = 0.5375, *SD* = 1.3213 - mechanical, interaction: *M* = 0.8149, *SD* = 2.2508 - mechanical, no interaction: *M* = -0.0581, *SD* = 1.1559 |
| Entity Type × Action Sequence Interaction in Right Hemisphere (Figure 11) | - NS | - social, interaction: *M* = -0.2096, *SD* = 0.3980 - social, no interaction: *M* = -0.0362, *SD* = 1.3760 - mechanical, interaction: *M* = -0.0955, *SD* = 1.1303 - mechanical, no interaction: *M* = 0.0099, *SD* = 0.9174 | - social, interaction: *M* = -0.0465, *SD* = 0.5428 - social, no interaction: *M* = 0.2435, *SD* = 1.0936 - mechanical, interaction: *M* = 0.7955, *SD* = 2.2889 - mechanical, no interaction *M* = -0.5120, *SD* = 1.0908 |
